# Comprehensive multi‐tissue epigenome atlas in sheep: A resource for complex traits, domestication, and breeding

**DOI:** 10.1002/imt2.254

**Published:** 2024-12-15

**Authors:** Deyin Zhang, Jiangbo Cheng, Xiaolong Li, Kai Huang, Lvfeng Yuan, Yuan Zhao, Dan Xu, Yukun Zhang, Liming Zhao, Xiaobin Yang, Zongwu Ma, Quanzhong Xu, Chong Li, Xiaojuan Wang, Chen Zheng, Defu Tang, Fang Nian, Xiangpeng Yue, Wanhong Li, Huibin Tian, Xiuxiu Weng, Peng Hu, Yuanqing Feng, Peter Kalds, Zhihua Jiang, Yunxia Zhao, Xiaoxue Zhang, Fadi Li, Weimin Wang

**Affiliations:** ^1^ State Key Laboratory of Herbage Improvement and Grassland Agro‐ecosystems; Key Laboratory of Grassland Livestock Industry Innovation, Ministry of Agriculture and Rural Afairs; Engineering Research Center of Grassland Industry, Ministry of Education; College of Pastoral Agriculture Science and Technology Lanzhou University Lanzhou China; ^2^ Lanzhou Veterinary Research Institute Chinese Academy of Agricultural Sciences (CAAS) Lanzhou China; ^3^ College of Animal Science and Technology Gansu Agricultural University Lanzhou China; ^4^ College of Science Gansu Agricultural University Lanzhou China; ^5^ Key Laboratory of Exploration and Utilization of Aquatic Genetic Resources, Ministry of Education Shanghai Ocean University Shanghai China; ^6^ Department of Genetics University of Pennsylvania Philadelphia PA USA; ^7^ Yazhouwan National Laboratory Sanya China; ^8^ Department of Animal Sciences and Center for Reproductive Biology Washington State University (WSU) Pullman WA USA; ^9^ Key Laboratory of Agricultural Animal Genetics, Breeding, and Reproduction of the Ministry of Education Huazhong Agricultural University Wuhan China

**Keywords:** multi‐omics, epigenomics, regulatory elements, genome‐wide association studies, *BMP2*, sheep

## Abstract

Comprehensive functional genome annotation is crucial to elucidate the molecular mechanisms of agronomic traits in livestock, yet systematic functional annotation of the sheep genome is lacking. Here, we generated 92 transcriptomic and epigenomic data sets from nine major tissues, along with whole‐genome data from 2357 individuals across 29 breeds worldwide, and 4006 phenotypic data related to tail fat weight. We constructed the first multi‐tissue epigenome atlas in terms of functional elements, chromatin states, and their functions and explored the utility of the functional elements in interpreting phenotypic variation during sheep domestication and improvement. Particularly, we identified a total of 753,723 nonredundant functional elements, with over 60% being novel. We found tissue‐specific promoters and enhancers related to sensory abilities and immune response that were highly enriched in genomic regions influenced by domestication, while *longissimus dorsi* tissue‐specific active enhancers and tail fat tissue‐specific active promoters were highly enriched in genomic regions influenced by breeding and improvement. Notably, a variant, Chr13:51760995A>C, located in an enhancer region, was identified as a causal variant for tail fat deposition based on multi‐layered data sets. Overall, this research provides foundational resources and a successful case for future investigations of complex traits in sheep through the integration of multi‐omics data sets.

## Highlights

- Generated 92 transcriptomic and epigenomic data sets from nine sheep tissues, establishing the first multi‐tissue epigenome atlas for functional genomic elements.
- Identified 753,723 nonredundant functional elements, with over 60% being novel, including tissue‐specific promoters and enhancers linked to sensory abilities, immune response, and tail fat deposition.
- Discovered a novel variant, Chr13:5176099A>C, located in an enhancer region, pinpointed as a causal variant for tail fat deposition using multi‐layered data sets.

## Graphical abstract

We constructed the first multi‐tissue epigenome atlas in sheep, identifying a total of 753,723 nonredundant functional elements, with over 60% being novel. Our findings highlight tissue‐specific functional elements, particularly those related to sensory abilities and immune response, which are highly enriched in genomic regions influenced by domestication. We identified the A allele of Chr13:51760995A>C as a favorable SNP through integrated analysis of multi‐omics datasets. Our study provides foundational data resources and a successful case for understanding the adaptive evolution and complex traits of sheep.

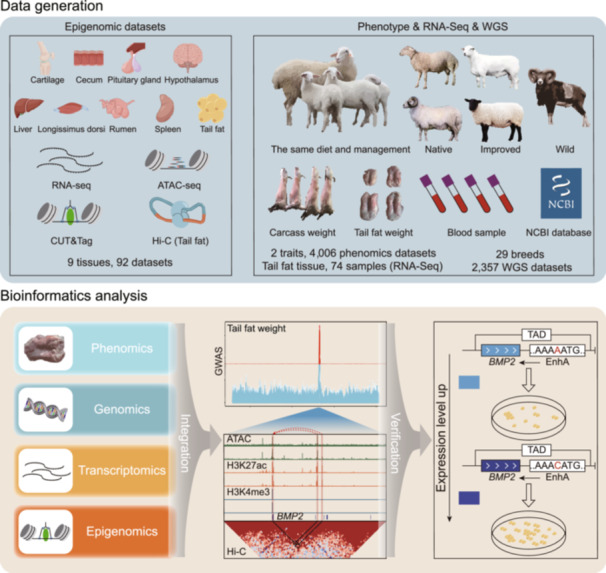

## INTRODUCTION

Sheep (*Ovis aries*), one of the most important agricultural animals, is an essential resource of meat, milk, and wool for humans [1]. Despite their economic importance, the genetic and molecular mechanisms underlying adaptive evolution and essential agronomic traits in sheep remain poorly understood. Genome‐wide association studies (GWAS) have been widely used to understand the genetic architecture of diseases and complex traits in humans and other livestock species [2, 3, 4, 5]. However, a major challenge in interpreting GWAS results is that many candidate loci associated with complex traits are located in noncoding regions of the genome, with many variants showing strong linkage disequilibrium (LD) [6, 7]. These factors severely hinder the understanding of the molecular mechanisms of phenotypic variation, adaptive evolution, and important agronomic traits in sheep.

By following the Encyclopedia of DNA Elements (ENCODE) and Epigenome Roadmap projects [8, 9], the Farm Animal Genotype‐Tissue Expression (FarmGTEx) project and the Functional Annotation of Animal Genomes (FAANG) initiative have significantly advanced the annotation of functional elements across various tissues in multiple livestock species, providing a rich resource for understanding the genetic mechanisms of complex agronomic traits [10]. However, the currently available annotations of regulatory elements in the sheep genome are limited to only a few tissues [11, 12], and the comprehensive annotation of functional elements in the sheep genome is lagging behind that of other animal species, such as pigs [13, 14, 15], cattle [16], chickens [17, 18], and model organisms [19, 20]. This limited annotation hinders our understanding of the molecular mechanisms underlying complex agronomic traits in sheep. Consequently, there is an urgent need to construct a comprehensive atlas of functional elements in the sheep genome to identify the candidate causative variants for economically important traits. Tail fat deposition, a major economic trait of fat‐tailed sheep breeds, has been understudied. Reducing tail fat deposition is essential for increasing the economic benefits of sheep farming. However, only a few key genes and functional mutations associated with tail fat deposition have been identified for us in breeding programs. Current research focuses primarily on easily measurable traits, such as tail size and configurations, but neglects the key indicator—tail fat weight [1, 21, 22]. Additionally, large quantitative trait loci regions identified through selection signals and GWAS are difficult to interpret due to the high number of variants. An integrated multi‐omics approach is needed to pinpoint causative variants for complex traits in livestock [23, 24], but such strategies have not yet been employed to study tail fat deposition in sheep.

In this study, we generated multiple layers of data sets, including 92 transcriptomic and epigenomic (Assay for transposase‐accessible chromatin using sequencing: ATAC‐seq, cleavage under targets and tagmentation: CUT&Tag and high‐throughput chromosome conformation capture: Hi‐C) data sets from nine major tissues, 2357 whole genome sequence samples from 29 breeds around the world and 4006 phenotypic data related to tail fat weight traits. We aimed to (1) build the multi‐tissue epigenome atlas, (2) explore the utility of functional elements in interpreting adaptive evolution and complex traits in sheep, and (3) identify candidate causal variants and their target gene contributing to tail fat deposition through integrated multi‐omic data. In summary, our research enriches the annotation of functional elements in the sheep genome and identifies causal variant affecting tail fat deposition, which provides foundational resources and a successful case study for future research on the biological mechanisms underlying complex economic traits in sheep.

## RESULTS

### Data summary

**FIGURE 1:**
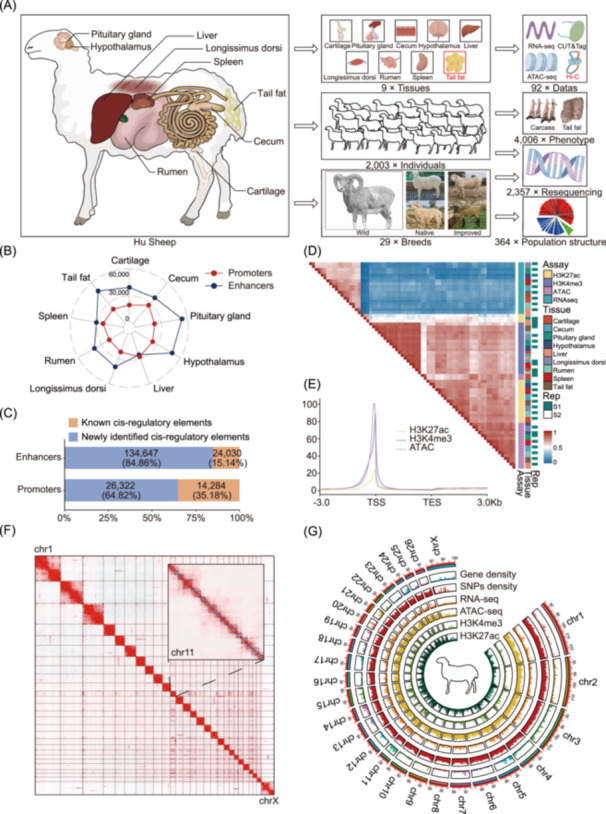
A multi‐tissue sheep epigenome atlas. (A) Schematic overview of tissues and data sets assayed in this study. Diagram of nine major tissue types from Hu sheep sampled (left), illustrations of tissue types, experiment population and representative breed (middle), sample number of epigenomic, phenotype, and genomics for the study (right). (B) The radar plot of promoters and enhancers across nine different sheep tissues. The red and blue lines represent the number of promoters and enhancers, respectively. (C) Percentages of promoters and enhancers were newly annotated in this study (blue) and overlapped with the previously published data for the sheep liver tissue (orange). (D) Heatmap for Pearson correlation of assays, tissues, and two biological replicates based on the normalized signal in 500 bp windows across the entire genome. (E) The distribution of average epigenetic mark signal around TSSs of genes. TSS, transcription start site; TES, transcription end site. (F) Heatmap of genome‐wide Hi‐C interaction matrices in sheep tail fat tissue. The rectangle in the upper right corner represents a magnification of chromosome 11, and the cyan dot and blue rectangle represent the identified loop and TAD, respectively. (G) Circos plot summarizing the chromosomal distribution of epigenetic marks. The outermost to innermost tracks represent ideograms of gene density, SNP density, RNA‐seq, ATAC‐seq, and CUT&Tag (H3K4me3 and H3K27ac) data for each chromosome. ATAC‐seq, assay for transposase‐accessible chromatin using sequencing; CUT&Tag, cleavage under targets and fragmentation; Hi‐C, high‐throughput chromosome conformation capture; RNA‐seq, RNA sequencing; SNP, single‐nucleotide polymorphism; TAD, topologically associating domain.

To advance the functional annotation of the sheep genome and explore the utility of enhanced genome annotation to explain the complex traits in sheep, we generated 92 epigenomic data sets of nine major tissues (cartilage, cecum, pituitary gland, hypothalamus, liver, *longissimus dorsi*, rumen, spleen, and tail fat) using the following methods: RNA sequencing (RNA‐seq), ATAC‐seq, CUT&Tag for two histone modifications (H3K27ac and H3K4me3), and Hi‐C (Figure 1A). We obtained 14.9 billion clean reads, with an average mapping rate of 93.16% (Table S1). The fraction of reads in peaks (FRiP), relative strand cross‐correlation coefficient (RSC), and normalized strand cross‐correlation coefficient (NSC) showed that our sequencing data were of sufficient quality for downstream analyses (Table S2).

To explore the utility of enhanced genome annotation to explain the complex traits in sheep, we integrated 364 whole‐genome sequencing (WGS) data for 29 breeds around the world for population structure and selection signal analysis. We also obtained carcass and tail fat weight phenotype data as well as WGS data from 2003 sheep. After variant calling and filtration, 21,741,684 single nucleotide polymorphisms (SNPs) were identified for further GWAS analysis (Table S3).

### Overview of sheep multi‐tissue epigenome atlas

In all nine tissues, we identified an average of 258,403, 20,945, and 52,552 peak sequences in the ATAC‐seq, H3K27ac, and H3K4me3 data; with average lengths of 692.92, 2479.90, and 2029.79 bp, respectively (Figure S1A–D), these peak sequences accounted for 3.38%, 1.82%, and 3.72% of the entire sheep genome, respectively (Table S2 and Figure S1E), and ATAC peaks were annotated to noncoding regions, while H3K27ac and H3K4me3 peaks were mainly annotated to the promoter region of the genes (Figure S1F). In addition, we identified a total of 753,723 nonredundant regulatory elements, including 40,606 putative promoters, 158,677 putative enhancers, and 846,058 open chromatin regions (OCRs) (Figure 1B), and over 84% and 64% of enhancers and promoters were newly identified in this study (Figure 1C), and we also validated that over 50.51% and 31.35% of our detected promoters and enhancers overlapped with promoters and enhancers identified from previously published data in the liver [25] (Figure S1G).

We assessed the relationships across different tissues and assays using gene expression and epigenetic marks data sets, the hierarchical clustering and principal component analysis (PCA) results clearly mirrored the sequencing assays, followed by tissue types and biological replicates (Figure 1D, Figure S2). The ATAC‐seq, H3K4me3, and H3K27ac data sets demonstrated a strong positive correlation with each other (mean *R* = 0.68), and three active regulatory marks peak were distributed in the regions around the transcription start sites (TSSs) of the genes, whereas they showed a weak positive correlation with the RNA‐seq data set (Figure 1E and Table S4). In addition, to profile the three‐dimensional genome architecture of the sheep genome, we performed in situ Hi‐C on tail fat tissue, as a representative tissue, of two sheep, and constructed a genome‐wide interaction map at the resolution 25 kb. The result indicated the closer the spatial distance between chromosomes, the higher was the degree of interaction, particularly in the same chromosome (Figure 1F). Overall, we used the aforementioned data sets to generate a high‐resolution, multi‐tissue epigenome map (Figure 1G), which enrich the annotation of functional elements in the sheep genome.

### Definition and characterization of chromatin states across multi‐tissues and species

**FIGURE 2:**
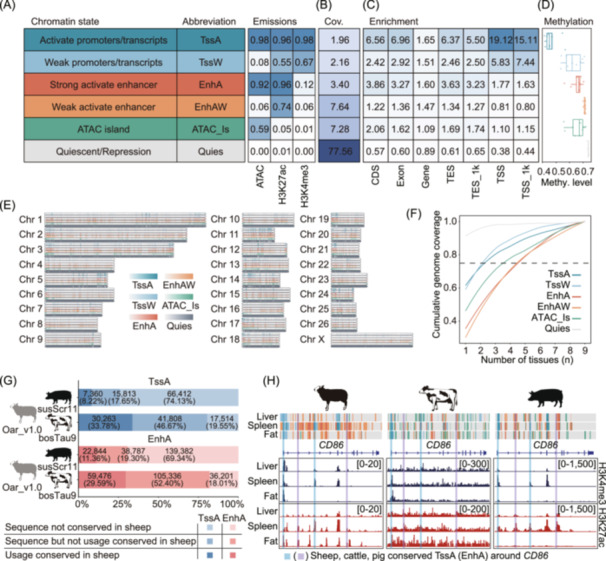
Genome‐wide chromatin state landscape across nine tissues. (A) Names, abbreviations, and emission probabilities of the chromatin states. (B) Genomic coverages of the chromatin states. (C) Average enrichment of different chromatin states in genome annotations. TSS/TES_1k, upstream and downstream 1 kb region of TSS and TES. (D) Average methylation level of chromatin states in liver. (E) Genome‐wide landscape of each chromatin state. TssA, activate promoters/transcripts; TssW, weak promoters/transcripts; EnhA, strong activate enhancer; EnhAW, weak activate enhancer; ATAC_Is, ATAC island; Quies, quiescent/repression. (F) Chromatin state variability based on cumulative genome coverage fraction. Dashed line = 0.75. (G) Sequence conservation and utilization of TssA and EnhA in the genomes of cattle and pigs, compared with sheep. (H) An example illustrating the conserved usage of TssA and EnhA at the *CD86* gene locus in sheep, cattle, and pigs. Numbers in brackets in the H3K4me3 and H3K27ac CUT&Tag tracks denote signal intensities.

We defined six chromatin states by integrating three epigenetic datasets (ATAC‐seq, H3K4me3, and H3K27ac) across nine tissues using the default parameters ChromHMM software. These chromatin states were mainly categorized promoter (TssA and TssW), enhancer (EnhA and EnhAW), ATAC island and quiescent/repression regions (Figure 2A,B). In general, we identified 1,936,312 nonredundant chromatin states across the nine tissues, the average lengths ranged between 906.88 and 25,332.1 bp, covering between 5.9% and 94.6% of the entire genome, respectively (Figure S3, and Table S5). The promoter states (TssA and TssW) exhibited the highest enrichment at TSS (Figure 2C), and also showed the highest average levels of gene expression than other chromatin states (Figure S4). In addition, we investigated the relationship between DNA methylation and chromatin states in matched tissues using publicly available DNA methylation data [25]. We found that activated promoter showed the lowest methylation level (Figure 2D), which confirms the well‐known negative correlation between gene expression and promoter methylation level [26, 27]. Simultaneously, a genome‐wide map of chromatin states was plotted on the basis of six nonredundant chromatin states across nine tissues. As expected, the repression regions are distributed in most regions of the entire genome, whereas the distribution of other chromatin states were similar (Figure 2E). In addition, we also examined the distribution of chromatin states in different tissues. The result showed that the activity of the promoter states (TssA and TssW) were less variable, while the activity of the enhancer states (EnhA and EnhAW) were highly variable between tissues (Figure 2F).

To further explore the conservation of chromatin states between the sheep, cattle, and pigs, we predicted six chromatin states from matched tissue types in these species using the same set of epigenetic marks, leveraging data from the FAANG and pig epigenome projects [14, 28]. Our analysis showed that the corresponding chromatin states across the three species exhibited similar emission probabilities for individual epigenetic marks (Figure S5). Additionally, we compared the sequence conservation of activate promoters (TssA) and strong active enhancers (EnhA) between the genomes of sheep, cattle, and pigs using LiftOver software (minMatch = 0.95). The results revealed that 80.45% of TssA and 81.99% of EnhA regions in the sheep genome were conserved in the cattle genome, while only 25.87% of TssA and 30.66% were conserved in the pig genome (Figure 2G). Moreover, both conserved and non‐conserved TssA and EnhA regions were observed near the orthologous *CD86* gene in sheep, cattle, and pigs, where consistent patterns of H3K4me3 and H3K27ac histone modifications were seen across the three species (Figure 2H). These findings suggest that regulatory elements tend to be more conserved between species with closer evolutionary relationships, and highly conserved regulatory elements may play similar functional roles across different species.

### Tissue‐specific analysis of genes and chromatin states

**FIGURE 3:**
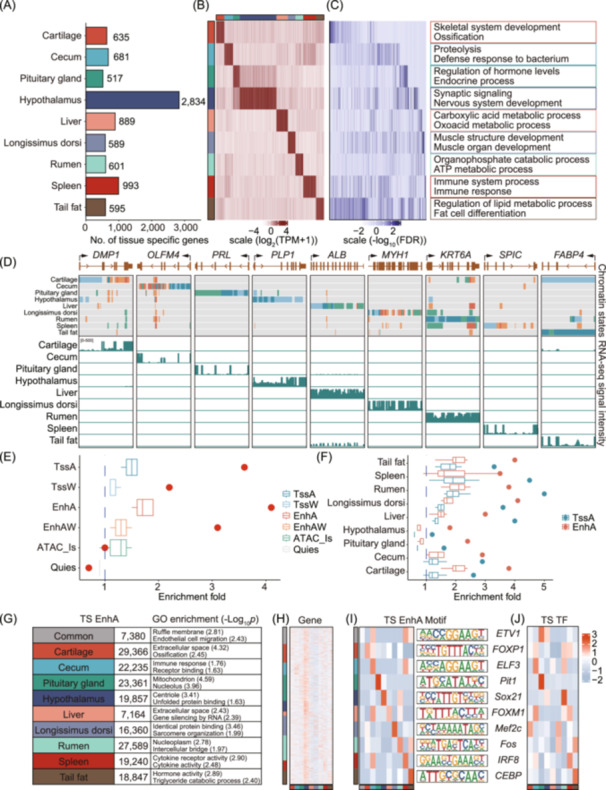
Functional characterization of tissue‐specific genes and chromatin states. (A–C) Gene numbers, expression pattern, and Gene Ontology (GO) enrichment terms of tissue‐specific genes (Tau > 0.8) across nine tissues. TPM, transcripts Per Million. (D) Example of chromatin state atlas in the context of tissue‐specific gene expression. For each gene locus, RNA‐seq signal tracks are shown across nine major tissues, with the vertical scale representing the normalized signal ranging from 0 to 500 for RNA‐seq. (E) Enrichment of *longissimus dorsi*‐specific genes in six chromatin states across nine tissues. The red dots represent chromatin states in *longissimus dorsi* tissue. (F) Enrichment of tissue‐specific genes for active chromatin states (TssA and EnhA) across nine tissues. The dots represent enrichments from matching tissues. The blue dashed lines indicate enrichment fold = 1. (G) Number of tissue‐specific (TS) EnhA, and GO enrichment of genes overlapping tissue‐specific EnhA. (H) Heatmap of expression patterns of genes overlapping tissue‐specific EnhA. (I) Motif enrichment in tissue‐specific EnhA of different tissues, and logos of their sequences. (J) Heatmap of transcription factor expression patterns corresponding with enriched motifs in different tissue‐specific EnhA.

Regulatory elements are important regulators for tissue‐specific gene expression. To investigate the relationship between functional elements and tissue‐specific gene expression, we detected a total of 8334 tissue‐specific genes across all nine tissues (Figure 3A,B and Table S6). The predicted functions of these tissue‐specific genes accurately reflected the specific biology processes of various tissues (Figure 3C and Table S7). Moreover, the quantitative real‐time reverse transcription PCR (qRT‐PCR)‐based gene expression analysis of representative examples of tissue‐specific genes verified the accuracy of our analysis (Figure 3D, Figure S6). We also observed that tissue‐specific genes exhibited a stronger enrichment of active chromatin states and a greater depletion of repressed regions in the corresponding tissues, compared to other tissues (Figure 3E,F). Simultaneously, we categorized genes into three groups based on the number of overlapping EnhA regions. Our analysis revealed that genes overlapping with a greater number of EnhA regions had higher expression levels and showed less pronounced tissue‐specific expression patterns compared to genes with fewer overlapping EnhA regions (Figure S7A). Gene ontology (GO) enrichment analysis demonstrated that genes with only one overlapping EnhA were associated with tissue‐specific functions, such as lysosome activity and phagocytosis in the spleen. In contrast, genes with multiple overlapping EnhA regions were enriched for more general biological functions (Figure S7B and Table S8).

To further explore the biological functions of active chromatin states, we identified 184,019 tissue‐specific EnhA across the nine tissues (Figure 3G) and found a significant positive correlation between tissue‐specific EnhA regions and tissue‐specific gene expression (*p* < 0.05) (Figure S8 and Table S9). The GO enrichment analysis results indicated that all genes overlapping with tissue‐specific EnhA showed known functions of the corresponding tissue (Table S10). Additionally, the genes overlapping with tissue‐specific EnhA were strongly expressed in not only the corresponding tissues but also the hypothalamus tissue (Figure 3H). We also conducted an enrichment analysis of motifs within tissue‐specific EnhA sequences and found the motif's expression levels were higher in the corresponding tissues compared to other tissues (Figure 3I,J). This indicates that tissue‐specific EnhA regions may regulate gene expression through the modulation of interactions between tissue‐specific transcription factors (TFs) and motifs, thereby influencing gene expression specificity. In addition, we explored the biological functions of tissue‐specific promoters. As expected, the number of TssA regions shared among all tissues exceeded the number of tissue‐specific TssA regions in most tissues, and the promoters also exhibited tissue‐specific functions (Figure S9), although to a lesser extent compared to EnhA regions, further support the crucial role of active chromatin states in regulating tissue‐specific functions. Overall, these results illustrate that the systemic analysis of chromatin states is important for downstream integration of epigenomic data to understand the genetic mechanisms underlying variation agronomic traits in sheep.

### Chromatin state predictions that enhance the understanding of phenotypic variation during sheep domestication and improvement

**FIGURE 4:**
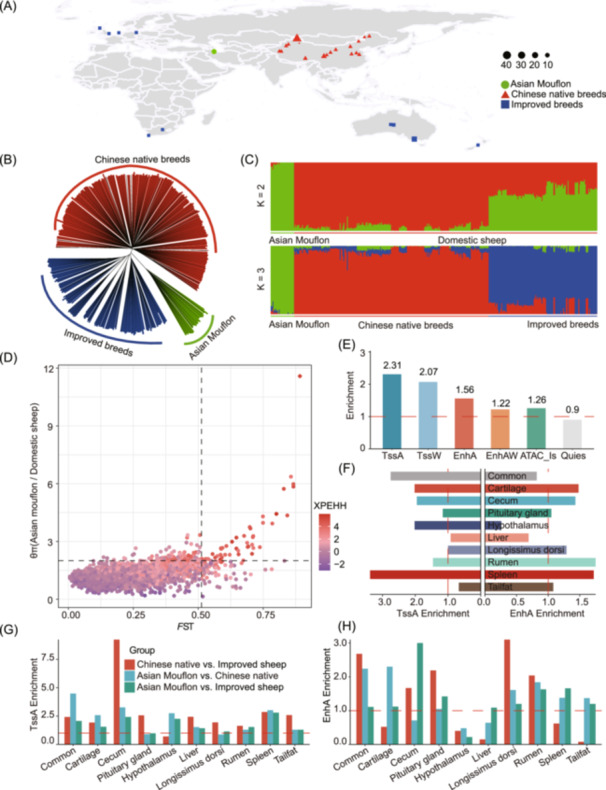
Enrichment of chromatin status for selected regions during sheep domestication and improvement. (A) Geographic distribution of Asian mouflon, Chinese native breeds, and improved breeds. The green circles, red triangles, and blue squares indicated Asian mouflon, Chinese native breeds, and improved breeds, respectively. The size of each point represents the number of samples. (B) Neighbor‐joining tree was constructed using p‐distance between individuals. (C) The population genetic structure of 364 individuals was inferred using ADMIXTURE. (D) Selective signature analysis comparing Asian mouflons with all the domestic sheep. The x‐axis shows fixation index (*F*_ST_ values), while the *y*‐axis represents nucleotide diversity ratios (θπ). XP‐EHH values are displayed using a color gradient. The threshold for significant selection signatures was set at the top 5‰ of outliers for each test. Black dashed lines in the upper‐right quadrant indicate the top 5‰ quantiles for all three statistics. (E) Enrichment of chromatin states within domestication selection signatures. (F) Enrichment of domestication selection signatures within tissue‐specific enhancers (EnhA) and promoters (TssA). (G, H) Enrichment of TssA and EnhA in selection signatures during domestication and breeding. Red dashed lines represent an enrichment fold of 1.

To explore whether genomic regions influenced by domestication and improvement are significantly enriched in functional elements. We first performed a population structure analysis based on the maximum likelihood estimation using 364 sheep from different counties (Figure 4A). The results showed that all individuals were classified into three separate clusters: Asian Mouflon, Chinese native sheep breeds, and improved breeds (Figure 4B,C, Figure S10). To dissect sheep domestication, we calculated the fixation index (*F*_ST_), population extended haplotype homozygosity (XP‐EHH), and the nucleotide diversity ratio (π ratio) between wild and domesticated sheep. In total, we identified 46 candidate genomic regions based on the top 5‰ windows across all three statistics (Figure 4D and Tables S11, S12). We found that TssA, TssW, and EnhA regions were highly enriched in genomic regions affected by domestication (Figure 4E). In our tissue‐specific chromatin state enrichment analysis, we observed the highest enrichment in spleen‐specific TssA and EnhA, followed by cecum‐specific TssA, both of which were linked to immune‐related functions, as well as hypothalamus‐specific TssA, linked to sensory functions (Figure 4F). This result is consistent with the fact that perceptual ability and survivability are preferentially selected in the earlier domestication process of sheep.

Additionally, we examined whether tissue‐specific TssA and EnhA were enriched in regions under selection by conducting pairwise comparisons of *F*_ST_, XP‐EHH, and π ratio between three sheep groups: Asian Mouflon vs Chinese native sheep breeds (WN), Asian Mouflon vs improved breeds (WI), and Chinese native sheep breeds vs improved breeds (NI). We identified 49, 53, and 13 candidate genomic regions in the WN, WI, and NI comparisons, respectively (Figure S11 and Tables S13–S18). Our analysis revealed significant enrichment of cecum, tail fat‐specific TssA, and *longissimus dorsi*‐specific EnhA in the NI group, while spleen, rumen, and tail fat‐specific EnhA were highly enriched in the WN and WI groups (Figure 4G,H). This is consistent with the breeding goals of improved sheep breeds, which focus on faster growth and reduced tail fat deposition, while native breeds prioritize adaptability and resilience. Overall, these results indicate the role of functional elements in explaining the adaptive evolution and complex agronomic traits in sheep.

### Identification of genomic regions associated with tail fat deposition in sheep

**FIGURE 5:**
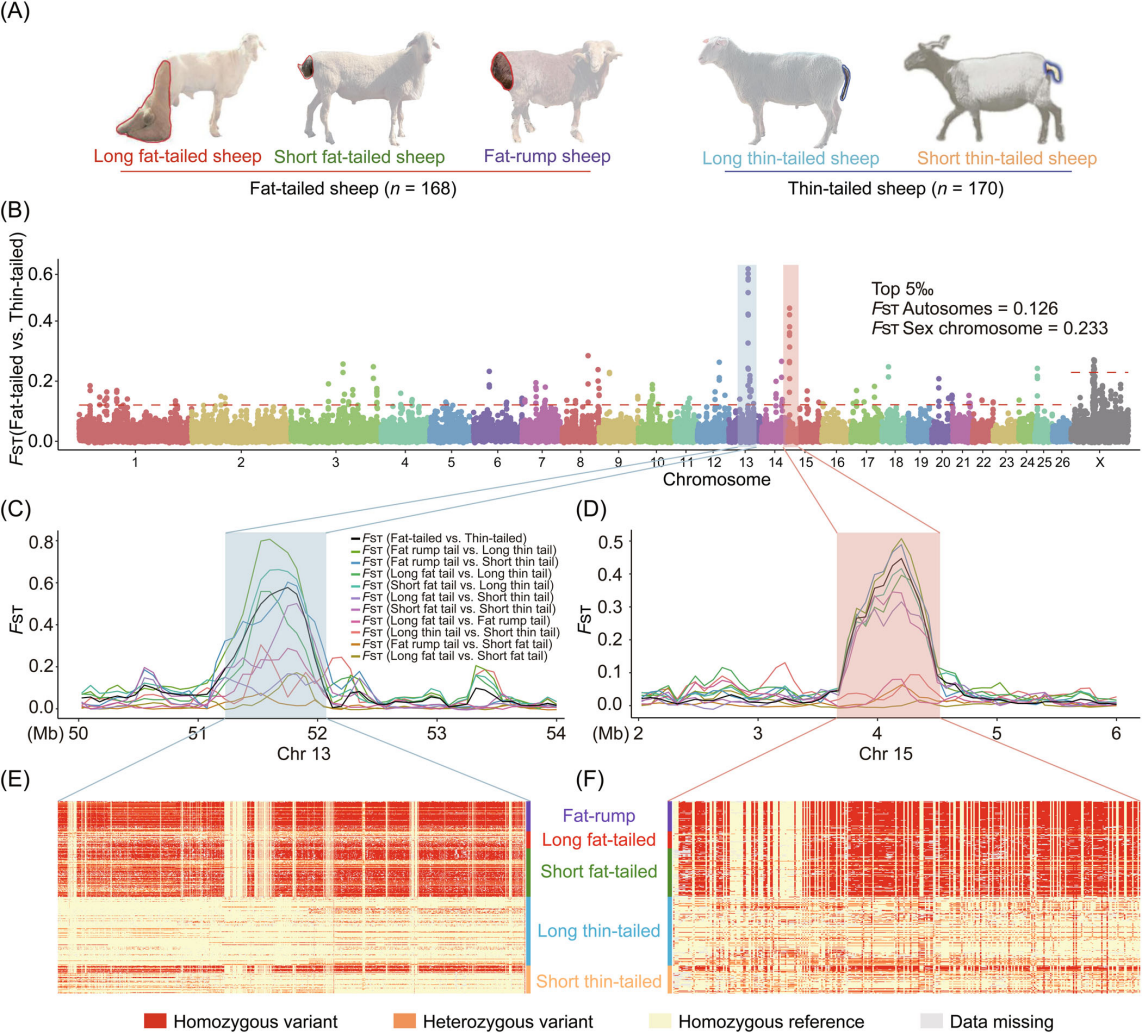
Genome‐wide selection sweep test of sheep with distinct tail morphology. (A) Illustrations of five distinct tail morphologies: long fat‐tailed, short fat‐tailed, fat‐rump, long thin‐tailed, and short thin‐tailed sheep. (B) Whole‐genome selective signals of thin‐ and fat‐tailed sheep breeds by *F*_ST_ analysis. The red dashed line indicates the top 5‰ threshold. (C, D) *F*_ST_ values plotted for selected regions using pairwise comparison of five tail morphologies on Chr13 C and Chr15 D. The different colored lines represent the *F*_ST_ values from pairwise comparisons of sheep with distinct tail morphology, with the black lines representing the *F*_ST_ values between the thin‐ and fat‐tailed sheep. (E, F) Haplotype differentiation patterns of different tail morphologies for the two most selected regions. Each row indicates one individual, and each column indicates one SNP. Red indicates homozygous variants, orange for heterozygous variants, yellow for homozygous reference and gray for missing data.

To identify the potential genomic regions associated with tail fat deposition in sheep, we compared the genomes of 168 fat‐tailed and 170 thin‐tailed sheep using the pairwise genetic differentiation (*F*_ST_) test (Figure 5A). The top 5‰ of windows with atypically high *F*_ST_ values (*n* = 189) were defined as the candidate selective regions. The two strongest signals were located on Chr13 (51.15–52.05 Mb) and Chr15 (3.67–4.50 Mb; Figure 5B and Table S19). We also performed pairwise population differentiation analysis by comparing long fat‐tailed, short fat‐tailed, fat‐rumped, short thin‐tailed, and long thin‐tailed breeds and assessed the same selected regions of Chr13 and Chr15 in a comparative analysis of the different fat‐ and thin‐tailed sheep populations (Figure 5C,D and Table S20). Further investigation of the allele frequencies in the two selected chromosome regions demonstrated that genotype patterns significantly differed between the thin‐ and fat‐tailed populations (Figure 5E,F), suggesting that these two regions possibly influence tail fat deposition.

**FIGURE 6:**
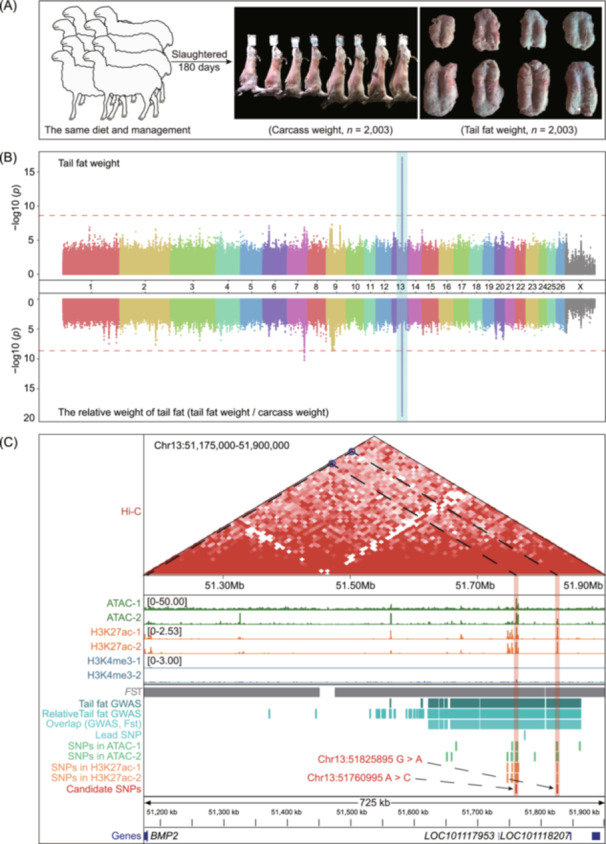
Integrating genome‐wide association studies (GWAS) and epigenomes data to locating candidate variant significantly associated with tail fat deposition in sheep. (A) Diagram of the tail fat weight (right) and carcass weight (middle) at 180 days of age in male Hu sheep (*n* = 2003). (B) Manhattan plots of GWAS for the tail fat weight and the relative weight of tail fat (tail fat weight/carcass weight) in the Hu sheep population (*n* = 2003). The dashed red line indicates the genome‐wide significance threshold [i.e., –log_10_ (0.05/total SNPs) = 8.63]. (C) Integrative analysis of multi‐omics data of tail fat deposition in sheep, showing the Hi‐C interaction heatmap of chr13:51,175,000–51,900,000 region at 10 Kb resolution, and the epigenetic signal of ATAC‐seq, CUT&Tag (K3K27ac and H3K4me3) in tail fat tissue, two replicates for each assay. The values in brackets (left) indicate ATAC‐seq and CUT&Tag (K3K27ac and H3K4me3) signal intensities. The track view of the variant, showing the selection region, GWAS region, lead SNP, ATAC‐seq, and CUT&Tag peaks, candidate SNPs, and nearby genes for the 51,175,000–51,900,000 bp region on Chr13.

To further narrow the genomic regions that influence tail fat deposition in sheep, we performed large‐scale GWAS for tail fat weight and relative weight of tail fat (tail fat weight/carcass weight) traits from the cohort of 2003 sheep (Figure 6A). On the basis of a threshold of −log_10_ (0.05/total SNPs) = 8.63, tail fat weight and relative weight of tail fat traits were linked to 984 and 1,086 significant SNPs, respectively, on Chr7, Chr9, and Chr13 (Figure 6B and Tables S21, S22), Of them, 981 SNPs overlapped and located in the selection sweep region of Chr13 (Table S23). Notably, these SNPs were located in intron and intergenic regions in a strong LD state (Figure S12), making it necessary to integrate multi‐omics data to determine the causal variant contributing to tail fat deposition.

### Prioritization of candidate causative mutations associated with tail fat deposition using multiple layers of datasets

To determine candidate causal variant and investigate the contributions of genomic variants to tail fat deposition, we integrated the results of the selective sweep analysis of different tail configurations, the GWAS results of the tail fat weight trait, and the epigenomic data (i.e., ATAC‐seq, H3K27ac, H3K4me3, and Hi‐C data) of sheep tail adipose tissue (Figure 6C). The results demonstrated that 71.3% of GWAS SNPs were located in the selected region on Chr13, whereas only 3.59% and 3.14% of the SNPs were located in the OCR and H3K27ac peaks of at least one individual. Notably, we only found that two SNPs (Chr13:51760995A > C and Chr13:51825895G > A) were located in the OCR and the H3K27ac peak. These two SNPs were also located in the same topologically associating domain (TAD) as the bone morphogenetic protein 2 (*BMP2*), *LOC101117953*, and *LOC101118027* genes (Figure 6C). These results demonstrated that these two SNPs may be related to sheep tail fat deposition.

**FIGURE 7:**
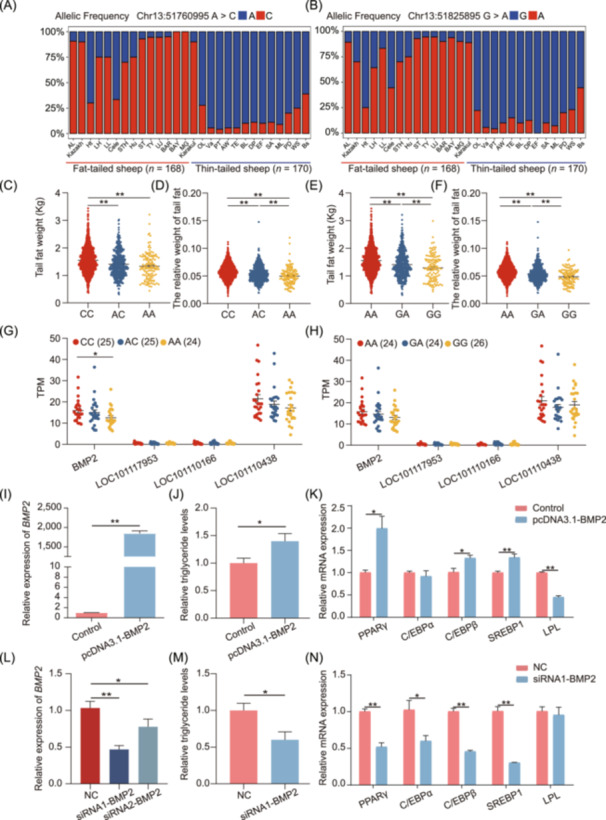
Identification of causal variant and verification of target genes. (A, B) Spectrum of allele frequencies at the Chr13:51760995A>C and Chr13:51825895G>A loci in 28 domestic sheep breeds. The type of reference allele is indicated in blue, while the mutant alleles in red. AL, Altay sheep; Ht, Hetian sheep; LH, Large‐tailed Han sheep; LL, Lanzhou Large‐tailed sheep; Cele, Qira Black sheep; STH, Small‐tailed Han sheep; ST, Short tail sheep; TY, Tan sheep; UJ, Ujimqin sheep; BAR, Barag sheep; BAY, Bayinbuluke sheep; MG, Mongolian sheep; OL, Tibetan sheep (Oula); Va, Tibetan sheep (Valley); PT, Tibetan sheep (Prairie); AW, Australian white; TE, Texel; BL, Border leicester; DP, Dorper; EF, East friensian milk sheep; SA, SA mutton merino; ML, Australian Merino; PD, Poll Dorset; WS, White suffolk; Bs, Black suffolk. (C–F) Tail fat weight and relative weight of tail fat of different genotypes at the Chr13:51760995A>C and Chr13:51825895G>A loci in the male Hu sheep population (*n* = 2003), respectively. (G, H) Expression level of candidate genes in different genotypes of the Chr13:51760995A>C and Chr13:51825895G>A loci. The red, blue, and orange dots are expressed as homozygous variants, heterozygous variant, and homozygous reference, respectively. (I) Overexpression efficiency of the pcDNA3.1‐BMP2 plasmid. (J) Triglyceride levels in preadipocytes transfected with the overexpression vector. (K) Adipogenesis marker gene expression levels in preadipocytes transfected with the overexpression vector. (L) Interference efficiency after the transfection of tail preadipocytes using the *BMP2*‐targeting siRNAs. (M) Triglyceride levels in preadipocytes transfected with the *BMP2*‐targeting siRNAs. (N) Adipogenesis marker gene expression levels in preadipocytes transfected with the *BMP2*‐targeting siRNAs. Data are indicated as means ± standard errors of the means; differences were analyzed by two‐tailed Student's *t*‐test. *p < 0.05, **p < 0.01.

To further explore how the candidate variants affect sheep tail fat deposition, we counted the allele frequencies at Chr13:51760995A>C and Chr13:51825895G>A SNPs in 338 domestic sheep with different tail configurations. The reference alleles Chr13:51760995 A and Chr13:51825895 G demonstrated higher frequencies in the thin‐tailed sheep breeds compared to the fat‐tailed sheep breeds (Figure 7A,B). The tail fat weight and relative weight of tail fat (tail fat weight/carcass weight) were significantly lower in the individuals with the reference allele (A/G) than in the individuals with the mutant alleles (C/A, *p* < 0.01, Figure 7C–F). Furthermore, tail fat transcriptome analysis of sheep with different genotypes demonstrated that only *BMP2* expression differed significantly between the individuals carrying the mutant alleles C allele and those carrying the reference allele A allele at Chr13:51760995A>C (*p* < 0.05). In contrast, the mutation in Chr13:51825895G>A did not affect the expression of *BMP2* and its nearby genes (Figure 7G,H). These results indicate that the alteration at the Chr13:51760995A>C SNP influences the expression of the *BMP2* gene.

To validate the effects of *BMP2* gene on sheep tail fat deposition, we isolate the primary preadipocytes from the tail adipose tissue and induced the differentiation into mature white adipocytes. We constructed an overexpression vector pcDNA3.1‐BMP2 and tested the overexpression efficiency, proliferation, and the differentiation potential. The results showed that the *BMP2* overexpression did not affect cell proliferation, while enhanced adipogenic potential, as indicated by the increased lipid accumulation and triglyceride content, as well as the elevated expression of adipocyte marker genes (Figure 7I–K, Figure S13A–C). In contrast, the lipid accumulation and triglyceride content in the transfected cells with siRNAs targeting the *BMP2* gene were significantly lower compared to those in the negative control group (*p* < 0.05) (Figure 7L–N, Figure S13D–F). Taken together, these results indicated that Chr13:51760995A>C, located in an enhancer region, is a compelling candidate causal variant that influences tail fat weight trait.

## DISCUSSION

Here, we generated and characterized the epigenomic landscape of functional elements across nine tissues. This represents the most comprehensive reference epigenomic data set for sheep reported to date, including 753,723 nonredundant regulatory elements and 8334 tissue‐specific genes. Notably, over 84% of the enhancers and 64% of the promoters identified were newly discovered compared to previous data [25]. Furthermore, we defined and characterized the chromatin states across nine tissues and performed the chromatin state comparisons among sheep, cattle, and pig. In addition, by integrating functional elements with selection signatures and large‐scale GWAS results, we explored the role of functional elements in understanding adaptive evolutions and tail fat deposition in sheep. This epigenomic landscape will provide essential resources for the annotation of the functional genome, comparative analysis across species, annotate and validate GWAS results, genome selection, and editing.

Elucidating the genetic mechanisms of adaptive evolution and complex traits in domestic animals is an active research area. Functional regulatory elements are crucial in the genetic regulation of adaptive evolution and complex traits [14, 15, 17]. Hence, we first explored the potential role of functional elements on sheep domestication and selection by focusing on the early stage of domestication. The results indicated that the genomic regions influenced by domestication are significantly enriched in functional regulatory elements, consistent with previously reported results [15]. Moreover, during domestication, the spleen specific TssA and EnhA were noted to be most enriched, followed by TssA specific to the cecum, and hypothalamus tissues. This result is consistent with previous findings that immunity and sensory ability may be important physiological phenotypes, preferentially selected during early domestication [29, 30]. In addition, during the improvement and breeding processes, the cecum‐specific TssA, rumen, and *longissimus dorsi*‐specific EnhA demonstrated the highest enrichment in the candidate selection signatures during the comparison of wild sheep or Chinese native breeds with improved breeds. This may be the result of the intensive selection of improved sheep breeds for growth rate and feed efficiency traits, native sheep breeds could be more adaptability and resilience. The rumen, a specialized organ found only in ruminants, is primarily responsible for digestion and nutrient absorption, and thus it is closely associated with feed efficiency in ruminants [31]. In addition, we found that the tail fat‐specific TssA was highly enriched in the candidate selection signatures during the comparison of Chinese native sheep with improved breeds. This result may reflect the observed distinct tail phenotypic differences between Chinese native sheep and improved breeds. Approximately 60% of Chinese native sheep breeds are fat‐tailed, whereas the improved sheep are mainly thin‐tailed. Tail fat deposition is a major economic trait that undergoes intensive selection during the improvement and breeding. Excessive tail fat deposition affects not only the mating and normal locomotion but also the meat quality, feed efficiency, and economic benefit in sheep [32, 33]. Thus, to determine the molecular mechanisms underlying tail fat deposition, researchers have exerted great efforts to identify candidate genomic regions or genes related to tail fat deposition [34, 35, 36, 37, 38]. However, these studies reported inconclusive results, possibly because they mainly used indirect indicators such as tail lengths and types and tail vertebra numbers as target traits with RNA‐seq, genome‐wide scans, and GWAS methods [1, 39, 40, 41]. Although a few studies have included tail fat weight as the target trait, they have used small sample sizes and considered small effect sizes for most candidate variants and high LDs between variants.

In the present study, we first performed a comprehensive selection signature analysis of 168 fat‐tailed and 170 thin‐tailed sheep worldwide. Then, for the first time, we performed GWAS on tail fat weight traits by using a large‐scale experimental population (*n* = 2003) and narrowed down the genomic region associated with tail fat deposition to Chr13. Despite narrowing down the genomic regions using comparative population genomics and GWAS, we could not identify causal variants and their target genes. Finally, through integrated analysis of selection signatures, GWAS results, epigenomic data of tail fat tissue, and RNA‐seq data, we found the A allele of Chr13:51760995A>C to be favorable SNP because it was associated with lower tail fat weight and decreased *BMP2* expression in tail fat tissues. BMP2, a transforming growth factor beta superfamily member, has important roles in adipogenic differentiation [42, 43]. Our functional verification results confirmed that *BMP2* overexpression can increase adipogenic potential, but its inhibited expression prevents adipogenesis. These results corroborate previously reported results [44, 45]. These results further suggest that the SNP Chr13:51760995A>C is a compelling candidate causal variant that influences tail fat weight trait.

Overall, this resource could assist identify candidate causal variants underlying agronomic traits in sheep, accelerating biology‐driven selective breeding to meet the demands of a growing global population. Notably, the SNP Chr13:51760995A>C, identified through the integration of multiple omics resources, could be of great interest, particularly in fat‐tailed sheep breeds. This result provides valuable molecular markers that could facilitate the breeding of sheep with reduced tail fat deposition or thin tail sheep through genomic selection or marker‐assisted selection, contributing to reduced animal suffering for tail docking and enhanced health and welfare. Despite the significant findings of this study, several limitations should be acknowledged. First, while we generated a multi‐tissue epigenome atlas from nine major tissues, it may not fully cover all biologically relevant tissues in sheep. Second, although the SNP Chr13:51760995A>C was identified as a candidate causal variant associated with tail fat deposition through integrative multi‐omics analysis, further functional validation—such as gene knockout or gene inhibition experiments—is needed to elucidate the precise role of Chr13:51760995A>C and *BMP2* in tail fat regulation. Additional validation across other sheep breeds or populations is also necessary to assess the generalizability of these findings.

## CONCLUSION

Our study established the first multi‐tissue epigenomic blueprint of sheep to fill a knowledge gap in existing sheep atlas datasets. This provides foundational data resources for understanding the adaptive evolution and complex economic traits of sheep. Simultaneously, through integration analysis of our multi‐layered data sets, we identified a novel causal SNP (Chr13:51760995A>C) that could serve as potential genetic marker for reducing tail fat deposition in sheep breeding programs. Overall, these findings provide valuable resources and molecular marker applicable to sheep breeding, helping to accelerate genetic improvement.

## METHODS

### Sample collection and data sources

To construct the multi‐tissue epigenome atlas, we collected 18 tissue samples (from the cartilage, cecum, pituitary gland, hypothalamus, liver, *longissimus dorsi*, rumen, spleen, and tail fat) from two half‐sib male Hu lambs aged 6 months.

For population structure and selection signature analysis, we obtained 364 genomic data sets, including the newly produced WGS data for 50 sheep from five breeds in this study (10 individuals from the population used for GWAS analysis), and WGS data of 314 individuals (26 Asiatic mouflons, 167 individuals from 13 Chinese native sheep breeds, and 121 individuals from 10 improved breeds) from our previous study [29] and publicly available databases (Table S24).

For GWAS, we constructed a comprehensive cohort comprising 2003 male Hu lambs and divided it into seven annual batches (from 2018 to 2022) for animal performance measurement and sample collection. All lambs of similar age were selected randomly from the National Core Breeding Farms of Sheep and Goats, as well as a substantial Hu sheep farm, right after they were weaned; they were transferred to the Minqin Experimental Farm of Lanzhou University and raised under the same management conditions and diet. When the lambs were aged 6 months, we collected their whole blood samples via jugular venipuncture; the blood samples were stored at −20°C until genomic DNA isolation. Next, each lamb was slaughtered to measure their carcass and tail fat weights. All tissue samples in this study were washed with ice‐cold phosphate‐buffered saline (PBS) and collected within 30 min post‐slaughter. The samples were immediately immersed in liquid nitrogen and transported to the laboratory. Finally, the samples were stored at −80°C in an ultralow‐temperature freezer until further use.

### Library construction and sequencing

For RNA‐seq, total RNA was isolated from nine tissues (two replicates for each tissue) using the FastPure Cell/Tissue Total RNA Isolation Kit V2 (RC112‐00, Vazyme), following the manufacturer's instructions. RNA purity and concentration were evaluated with a NanoDrop 2000 spectrophotometer (Thermo Fisher Scientific, USA), and only high‐quality RNA samples were used to construct strand‐specific RNA‐seq libraries. Library quality was assessed using an Agilent 2100 Bioanalyzer (Agilent Technologies), and sequencing was performed on the Illumina NovaSeq 6000 platform at Wuhan Yingzi Gene Technology.

For ATAC‐seq, approximately 5 mg of the frozen tissue sample was pulverized in liquid nitrogen and homogenized into cell suspensions in 1 mL of ice‐cold PBS. Following the previously described method with minor modifications [14], we extracted the nuclei and incubated them with the Tn5 transposase reaction mix at 37°C for 1 h; this was followed by purification using a DNA Purification and Concentration Kit (TD413; Genstone Biotech). The transposed DNA fragments were then amplified through PCR using a NEBNext High‐Fidelity 2X PCR Master Mix (M0541L; NEB), followed by purification using Kapa Pure Beads (KS8002; Kapa Biosystems) and sequencing on the Illumina NovaSeq. 6000 platform at Wuhan Yingzi Gene Technology.

For CUT&Tag, nuclei were extracted from each flash‐frozen tissue sample using the same method used for ATAC‐seq. The extracted nuclei were mixed with concanavalin A‐coated magnetic beads (BP531; BioMag Plus) and incubated at room temperature for 20 min. This was followed by incubation with the primary antibodies (anti‐H3K27ac, anti‐H3K4me3, and IgG). After incubation at room temperature for 1 h, the excess primary antibodies were washed off and incubated with a secondary antibody (goat anti‐rabbit IgG; ab6702; Abcam) at room temperature for 1 h, followed by three washes with DIG Wash Buffer. Next, protein G–Tn5 complex (Hyperactive pG‐Tn5 Transposase for CUT&Tag; S602; Vazyme) was added to the above system. After incubation at room temperature for 1 h, the reaction system was washed three times with 1× Dig‐300 Buffer (TD901‐TD902; Vazyme) and incubated in an Mg^2+^ activation system (AM9530G; Invitrogen) at 37°C for 1 h. Finally, sodium dodecyl sulfate (SDS) Buffer (15553‐027; Invitrogen) was used to stop the tagmentation reaction, and DNA was extracted using Tagment DNA Extract Beads (N245; Novoprotein) and then amplified using NEBNext High‐Fidelity 2X PCR Master Mix (M0541L; NEB). Finally, the libraries were purified using Kapa Pure Beads (KS8002; Kapa Biosystems) and then sequenced on the Illumina NovaSeq 6000 platform by Wuhan Yingzi Gene Technology.

For Hi‐C sequencing, the tail fat tissue samples were selected and processed according to a previously described method with minor modifications [14]. Briefly, approximately 1 g of a sample was pulverized and fixed with 1% formaldehyde (252549; Sigma‐Aldrich) at room temperature for 20 min. The reaction was then quenched with 0.125 M glycine solution (V900144; Sigma) by incubating at room temperature for 5 min. The crosslinked tissue samples were mixed with Hi‐C lysis buffer and incubated on ice for 15 min. Subsequently, 0.3% SDS was added, and the mixture was incubated at 62°C for 10 min, The reaction was then halted by adding Triton X‐100 (28314; Thermo Fisher Scientific). Next, chromatin was digested with *Alu*I endonuclease (R0137L; NEB) at 37°C for 7 h, followed by the addition of dATP (N0440s; NEB) and incubation at 37°C for 1 h. DNA ligation was performed using T4 DNA ligase containing a biotin linker (M0202L; NEB), followed by incubation at 16°C overnight. Finally, DNA was extracted, purified, and sequenced on Illumina NovaSeq. 6000 platform.

For WGS, genomic DNA of the sequenced samples was extracted from 2053 blood samples by using an EasyPure Blood Genomic DNA Kit (TransGen Biotech) following the manufacturer's instructions. DNA integrity and quality were assessed through agarose gel electrophoresis and a NanoDrop 2000 spectrophotometer (Thermo Fisher Scientific), respectively. Paired‐end sequencing libraries were constructed for each DNA sample and sequenced on the Illumina NovaSeq. 6000 platform (Novogene Co., Ltd.).

### Raw sequencing data analysis

In total, 2135 new data sets—including data sets from RNA‐seq (*n* = 18), ATAC‐seq (*n* = 18), CUT&Tag (H3K27ac, H3K4me3, and IgG) for nine tissues (*n* = 54), Hi‐C from tail fat tissue (*n* = 2), and whole‐genome sequences (*n* = 2043)—were generated and analyzed. We also uniformly analyzed another 314 whole‐genome sequence data sets and three liver whole‐genome bisulfite sequencing (WGBS) data from a previous study [25]. The detailed analysis method is as follows:

For RNA‐seq data, we used TopHat (version 2.1.1) [46] for alignment with default parameters; then, we applied Samtools (version 1.12) to sort the BAM file and quantified the read numbers mapped onto each gene by using featureCounts (version 2.0.3) [47]. Next, we normalized the raw count matrix to transcripts per million (TPM) for subsequent analysis. Tissue‐specific expressed genes among these were identified according to the previously described tau index method [48]; genes with tau values > 0.8 were defined as tissue‐specific genes [49].

For ATAC‐seq and CUT&Tag data, the sequencing reads were aligned using Bowtie2 (version 2.2.4) [50]; moreover, Samtools (version 1.12) [51] and Picard (version 3.1.1) were used to remove low‐quality mapped reads, unmapped reads, and PCR duplicates. The mitochondrial reads were removed using bedtools (version 2.26.0) [52]. Then, the peak for each replicate was called individually using MACS2 (version 2.2.6) [53], and the significant peak (*p* < 0.00001) was used for further analysis. Each significant peak was further annotated to the sheep reference genome (Oar_rambouillet_v1.0) using the R package ChIPseeker (version 1.34.1) [54]. In addition, the BAM files for two biological replicates were merged using Samtools, and the merged BAM files were further converted to bigWig files by bamCoverage (in deepTools), followed by visualization with the Integrative Genomics Viewer browser and deepTools (version 3.5.0). Finally, to analyze the correlation of read distributions among RNA‐seq, ATAC‐seq, and CUT&Tag data across different tissues, we converted the BAM files to BED files using bedtools (version 2.27.1). Whole sequences were partitioned into 500‐bp windows, followed by correlation analysis using Pearson coefficients. The results were visualized with the R package ComplexHeatmap.

For Hi‐C data, the clean reads from different libraries were aligned and filtered with the HiC‐Pro (version 3.1.0) [55] pipeline. Then, we merged the multiple libraries, generated contact matrices, and performed matrix‐balancing iterative correction and eigenvector decomposition (ICE) normalization. To investigate genome‐wide chromatin interactions, Juicebox (version 1.11.08) was used to build and visualize the contact matrices with 25‐kb resolution. HiCExplorer (version 3.7.2) [56] was applied for detecting significant TADs, with a significance threshold set to a false discovery rate of 0.05, and the corresponding interaction heatmaps and TAD boundaries were visualized using fancplot function of fanc (version 0.9.28).

For the WGBS data, raw reads were filtered using the trim_galore with default parameters (version 0.6.10). The clean reads were aligned to the reference genome using Bismark (version 0.24.2) and PCR duplicates were removed using picard MarkDuplicates (version 2.27.5). The methylation level of CpG site were extracted with the bismark_methylation_extractor function of bismark (version 0.24.2). The average methylation level for different chromatin state was then calculated with the bedtools map function (version 2.27.1).

For the WGS data, the filtered reads were mapped onto the sheep genome (Oar_rambouillet_v1.0) by using Burrows–Wheeler aligner (version 0.7.15) with default parameters. We converted the obtained mapping files to BAM files and sorted them by using Samtools (version 1.10) and removed PCR duplicates by using both Samtools and Picard. SNP calling was performed using the HaplotypeCaller function in Genome Analysis Toolkit (GATK), and the genomic variant call format (gVCF) file for each individual was obtained. Next, all the gVCF files were combined using CombineGVCFs, and SNPs were joint‐called using GenotypeGVCFs in GATK. Subsequently, VCFTools (version 0.1.16) was used to filter the SNP data sets by using the following parameters: –remove‐indels, –minDP 5, –min‐alleles 2, –max‐alleles 2, –maf 0.05, and –max‐missing 0.8. After all SNPs were filtered, they were annotated using ANNOVAR based on the gene annotations of the sheep reference genome (Oar_rambouillet_v1.0) and classified as variations in the exonic (i.e., synonymous or nonsynonymous SNPs), intronic, intergenic, upstream, and downstream regions, as well as those in splicing sites.

### Chromatin state partitioning

ChromHMM (version 1.24) [57] was used to perform an integrated analysis of CUT&Tag (H3K27ac and H3K4me3) and ATAC‐seq data set across nine tissues; the chromatin state of the sheep genome was annotated based on a multivariate hidden Markov model [58, 59]. Because six states can represent the most combinations of three epigenetic marks, we predicted six chromatin state models based on the enrichment of epigenetic modifications: active promoters (TssA), weak promoters (TssW), strong activate enhancer (EnhA), weak activate enhancer (EnhAW), ATAC island (ATAC_Is), and quiescent/repression (Quies). The fold enrichment degree of different chromatin state and genomic region was calculated with the OverlapEnrichment parameter [15].

### Conservation analysis of chromatin states across species

We downloaded the H3K27ac and H3K4me3 ChIP‐seq, and ATAC‐seq data for cattle (liver, spleen, fat, hypothalamus) and pig (skeletal muscle, liver, spleen, fat) from FAANG [28] and published articles [14]. The cattle and pig data were mapped to the susScr11 and bosTau9 reference genomes, respectively. All data processing and chromatin state partitioning were carried out following the same methods described above for sheep. To explore chromatin state conservation across species, the TssA and EnhA in cattle/pig genomic (bosTau9/susScr11) were converted to sheep genomic locations (Oar_rambouillet_v1.0) by using the LiftOver tool with the default parameter (minMatch = 0.95) [60]. The TssA and EnhA successfully converted sheep genomic coordinates were regarded as sequence‐conserved. Additionally, they were classified as usage‐conserved if their corresponding pig/cattle homologous sequences overlapped with pig/cattle TssA and EnhA.

### Identification of tissue‐specific chromatin states

To explore the tissue specificity of chromatin states, we used the merge parameter in bedtools and generated nonredundant chromatin states in nine sheep tissues. Then, we mainly performed the specific analysis of TssA and EnhA, overlapping the chromatin states of different tissues with nonredundant chromatin states. If the intersecting region was ≥ 1, the nonredundant chromatin state of the tissue was calculated as 1; otherwise, it was considered 0. A nonredundant chromatin state of 1 in a single tissue was considered a tissue‐specific chromatin state, and a nonredundant chromatin state of 1 in all tissues was regarded as a common chromatin state [61]. GO functional enrichment analysis was performed on genes overlapping with tissue‐specific regulatory elements. HOMER (version 4.11) was employed to identify motifs that are significantly enriched in tissue‐specific regulatory elements, and the motifs with the highest *p*‐value were demonstrated using the R package ComplexHeatmap. Motif‐binding TF expression was analyzed according to the expression matrix obtained using RNA‐seq.

### Tissue‐specific gene expression analysis

To identify tissue‐specifically expressed genes, we determined the tissue specificity of genes by calculating the Tau value of each gene according to the method described in the previous literature [48]. The calculation method of Tau value is as follows, where *x* represents the TPM value of tissue gene expression and *n* refers to the number of tissues.

$$\tau =\frac{\sum_{i=1}^{n}\left(1-{\hat{x}}_{i}\right)}{n-1},$$

$${\hat{x}}_{i}=\frac{x_{i}}{\max_{1\leq i\leq n}{(x}_{i})}.$$

The tissue‐specific gene clusters were visualized using the R package ComplexHeatmap (version 2.14.0), and GO enrichment analyses for tissue‐specific genes were performed on the DAVID website (https://david.ncifcrf.gov/).

### Population structure analysis

To investigate the population structure of all wild and domestic sheep, we converted the VCF files to the PLINK files by using VCFTools (version 0.1.16) and implemented pruning of autosomal SNPs using the –indep‐pairwise 50 5 0.2 parameters in PLINK (v. 1.9). The unlinked SNPs were used for phylogenetic tree reconstruction, population genetic structure, and PCA. The pairwise genetic distance matrix was calculated using PLINK and employed for constructing and visualizing a neighbor‐joining tree with the online tool iTOL (https://itol.embl.de/). Population structure analysis was performed using ADMIXTURE with the default settings. The numbers of assumed ancestral populations (K) were set at 2 and 3. Furthermore, PCA was performed using PLINK, and the plots of the first and second eigenvectors were created using the R package ggplot.

### Selection signature enrichment analysis of chromatin states

To identify putative selective regions during domestication and breeding, we performed fixation index (*F*_ST_), cross‐population extended haplotype homozygosity (XP‐EHH), and nucleotide diversity ratio (π ratio) analysis with a 150‐kb sliding window and 75‐kb step. First, *F*_ST_ values were calculated using VCFTools (version 0.1.16) for population pair comparisons (Asian mouflon vs domestic sheep, Asian mouflon vs Chinese native breed, Asian mouflon vs. improved breed, Chinese native breed vs improved breed). π values were calculated for each population using VCFTools (version 0.1.16), and the π ratio was calculated with an R script. XP‐EHH analysis was performed using Beagle (version 5.3) for haplotype inference, and XP‐EHH values between population pairs were calculated using Selscan (version 2.0.3) [62]. The results were normalized to obtain average XP‐EHH scores. The top 5% overlapping windows from all three methods were considered candidate selection signatures and subsequently used for chromatin state enrichment analysis. Fold enrichment of selection signatures for different chromatin state was calculated using a previously reported method [15]. We also implemented selective sweep tests between specific populations of domestic sheep with varied tail configurations; we specifically compared populations with fat‐ and thin‐tailed sheep, followed by pairwise comparisons of five tail configurations.

## GWAS

GWAS of tail fat weight and the relative weight of tail fat (tail fat weight/carcass weight) traits were performed in a cohort of 2003 sheep by using the mixed linear model method in the R package rMVP (https://github.com/xiaolei-lab/rMVP). In the association model, the birthplace, batch, rearing season, and top 3 principal components were considered fixed effect and covariates, the additive genetic effect was considered random effect. After Bonferroni correction, the genome‐wide significant threshold was set at –log_10_ (0.05/total SNPs) = 8.63.

### Transcriptome sequencing and analysis

In total, 74 tail tissue samples were randomly obtained from a comprehensive cohort comprising 2,003 male Hu lambs according to the genotypes at the Chr13:51760995A>C and Chr13:51825895G>A loci (Oar_rambouillet_v1.0). By using the method mentioned above, we extracted total RNA from each tissue and evaluated the RNA quality. Then, RNA‐seq libraries were prepared and sequenced on the Illumina NovaSeq 6000 platform by Shanghai Personal Biotechnology. The TPM value for each individual was obtained using the filtering and analysis methods mentioned above, and the candidate gene expression for the different genotypes at the Chr13:51760995A>C and Chr13:51825895G>A loci were measured and visualized using Prism (version 8.0.1; GraphPad).

### *BMP2* **siRNA interference and overexpression**

Two pairs of small interfering RNAs (siRNAs), synthesized by Maya Biotechnology, were used for *BMP2* knockdown (Table S25). Based on its efficiency, the si‐BMP2‐1 sequence was selected for subsequent analysis. To construct a *BMP2* overexpression vector, the forward and reverse primers containing *Bam*HI and *Eco*RI sites were designed based on the *BMP2*‐coding region sequence obtained through cloning, respectively. The amplified fragment was ligated with linear pcDNA3.1. The siRNA and primer sequences used in this study are listed in Table S25.

### Cell isolation and cultivation

Sheep preadipocytes were isolated from the tail fat tissue of 20‐day‐old Hu sheep and then purified and cultured following a previously described method [63] with minor modifications. Briefly, the tail fat tissue was cut into approximately 1‐mm^3^ blocks and minced; next, preadipocytes were obtained through digestion with type I collagenase and maintained in a basal medium containing 10% fetal bovine serum (FBS; Hyclone) and a 1% antibiotic–antimycotic solution. The cells were suspended in a 25‐cm^2^ culture flask and incubated at 37°C under 5% CO_2_. The medium was replaced with fresh medium after 24 h; thereafter, the medium was replaced with fresh medium every 48 h. When the primary preadipocytes reached approximately 80% confluence, they were digested with trypsin and passaged at a ratio of 1:3; finally, the third generation of preadipocytes was utilized for subsequent experiments.

### Cell counting kit‐8 (CCK‐8) assay for preadipocyte proliferation

Sheep tail preadipocytes were added at 1 × 10^3^/well in 96‐well plates and then cultured for 24 h. Next, the cells were treated with the *BMP2* overexpression vector or siRNA, followed by induction for differentiation in a differentiation medium (10% FBS, 1 μM dexamethasone, 0.5 mM 3‐Isobutyl‐1‐methylxanthine, and 10 mg/mL insulin) for 48 h. Cell proliferation was detected using the CCK‐8 kit at 450 nm on a microplate reader (Model 680).

### Oil red O staining

After transfection with the *BMP2* overexpression vector or siRNA, the preadipocytes were washed three times with PBS and fixed with 10% formaldehyde for 60 min. Next, we examined lipid accumulation by staining the cells with an oil red O staining kit, according to the manufacturer's instructions. Morphological changes in stained cells were observed and imaged under an inverted fluorescent microscope. Thereafter, isopropanol was added to extract the lipid droplet contents; the optical density (OD) of the developed color, reflecting the lipid droplet content, was measured at 510 nm.

### Cellular triglyceride content analysis

The preadipocytes were transfected with overexpression vectors or siRNAs, collected after 48 h of differentiation, and washed three times with precooled PBS. The cell triglyceride contents were assessed using a triacylglycerol assay kit (Nanjing Jiancheng Bioengineering Institute). The OD of the developed color was measured on a microplate reader (Bio‐Rad); the values were then normalized to the total protein contents (in µg/mg).

### RNA extraction and quantitative real‐time reverse transcription PCR (qRT‐PCR)

Total RNA was isolated using AG RNAex Pro Reagent (Accurate Biotechnology (Hunan) Co., Ltd.), following the manufacturer's protocol. RNA integrity and purity were assessed through agarose gel electrophoresis and quantified with a NanoDrop 2000 spectrophotometer (Thermo Fisher Scientific). Next, cDNA synthesis was performed using an Evo M‐MLV RT Kit with gDNA Clean for qPCR (Accurate Biotechnology, Hunan, China), according to the manufacturer's instructions. qRT‐PCR was performed using synthesized primers and an SYBR Green Premix Pro Taq HS qPCR Kit (Accurate Biotechnology). PCR conditions were similar to those described previously [64]. The relative expression levels of target genes were calculated using 2^−ΔΔCt^ method [65], with *UXT* and *ACTB* serving as the internal control genes. The primer sequences used here are provided in Table S25.

### Statistical analysis

Statistical analyses and data visualization were conducted using R software (version 4.2.1) and GraphPad Prism 8.0.1 (GraphPad Software). Pearson correlation between tissues, biological replicates, and assays was calculated using the cor() function in R. Group differences were assessed using either two‐tailed Student's *t*‐test or one‐way analysis of variance (ANOVA). Statistical significance was denoted by the following symbols: *p ≤ 0.05; **p ≤ 0.01; ***p ≤ 0.001; ****p ≤ 0.0001. Results were presented as mean ± SEM.

## AUTHOR CONTRIBUTIONS

**Deyin Zhang**: Conceptualization; investigation; writing—original draft; writing—review and editing; visualization; validation; software; formal analysis; data curation. **Jiangbo Cheng**: Conceptualization; investigation; writing—review and editing; visualization; validation. **Xiaolong Li**: Conceptualization; investigation; data curation; software; formal analysis. **Kai Huang**: Validation; visualization; investigation; data curation. **Lvfeng Yuan**: Data curation; software; formal analysis; methodology. **Yuan Zhao**: Data curation; methodology; software; formal analysis. **Dan Xu**: Formal analysis; data curation; validation; visualization. **Yukun Zhang**: Methodology; software; formal analysis; data curation. **Liming Zhao**: Data curation. **Xiaobin Yang**: Data curation; formal analysis. **Zongwu Ma**: Validation; data curation. **Quanzhong Xu**: Data curation; formal analysis. **Chong Li**: Data curation; validation; visualization. **Xiaojuan Wang**: Validation; visualization; data curation. **Chen Zheng**: Data curation; validation; visualization. **Defu Tang**: Data curation; visualization. **Fang Nian**: Data curation. **Xiangpeng Yue**: Data curation; resources. **Wanhong Li**: Data curation; resources. **Huibin Tian**: Data curation; resources; validation; visualization. **Xiuxiu Weng**: Data curation; resources. **Peng Hu**: Resources; writing—review and editing. **Yuanqing Feng**: Writing—review and editing. **Peter Kalds**: Writing—review and editing. **Zhihua Jiang**: Writing—review and editing. **Yunxia Zhao**: Writing—review and editing. **Xiaoxue Zhang**: Writing—review and editing; conceptualization; data curation; resources; validation; visualization. **Fadi Li**: Funding acquisition; resources; data curation; project administration; supervision. **Weimin Wang**: Software; formal analysis; project administration; resources; data curation; conceptualization; investigation; funding acquisition; writing—original draft; writing—review and editing; supervision.

## CONFLICT OF INTEREST STATEMENT

The authors declare no conflicts of interest.

## ETHICS STATEMENT

The ethics application (No. GSAU‐Eth‐AST‐2022‐022) was approved by the Animal Care Committee at Gansu Agricultural University and the Animal Care and Use Committee of Lanzhou University.

## Supporting information


**Figure S1:** Overview of data quality for epigenomic data sets.
**Figure S2:** Principal component analysis (PCA) of different data set types for nine sheep tissues.
**Figure S3:** Characterization of genome‐wide chromatin state across nine tissues.
**Figure S4:** Average gene expression level of different chromatin states across nine tissues.
**Figure S5:** Chromatin states predicated from matched tissue types in sheep, cattle and pigs.
**Figure S6:** Quantitative real‐time reverse transcription PCR (qRT‐PCR) plots in nine tissues of representative tissue‐specific genes across nine tissues.
**Figure S7:** The gene expression, tissue‐specific value (Tau), and GO terms for the three gene groups.
**Figure S8:** Correlation analysis between tissue‐specific gene expression and tissue‐specific enhancers.
**Figure S9:** Tissue‐specific (TS) activate promoters (TssA) and their potential functions across nine tissues.
**Figure S10:** Principal component analysis (PCA) of 364 individuals.
**Figure S11:** Genome‐wide selective signal analysis in different sheep populations.
**Figure S12:** Linkage disequilibrium (LD) analysis of overlapping SNPS for the tail fat weight and relative weight of tail fat (tail fat weight/carcass weight) trait.
**Figure S13:** Functional verification of the *BMP2* gene at cell level.

**Table S1:** Overview of sheep RNA‐seq, ATAC‐seq, CUT&Tag, and Hi‐C data.
**Table S2:** Peak statistics of ATAC‐seq and CUT&Tag.
**Table S3:** Distribution of SNPs within different genomic regions.
**Table S4:** Pearson correlation values of assays, tissues, and two biological replicates.
**Table S5:** The quantity, size, and gene coverage of nonredundant chromatin states.
**Table S6:** List of tissue‐specific genes.
**Table S7:** GO enrichment of tissue‐specific genes.
**Table S8:** GO enrichment analysis for genes overlapping with different EnhA numbers in the spleen tissue.
**Table S9:** The Pearson correlation analysis of the tissue‐specific gene expression and tissue‐specific enhancer.
**Table S10:** GO enrichment analysis of genes overlapping with tissue‐specific EnhA.
**Table S11:** Putative selected regions in Asian mouflon‐to‐domestic sheep comparison based on top 5‰ *F*_ST_, θπ, and XPEHH.
**Table S12:** The overlapping regions in Asian mouflon‐to‐domestic sheep based on 5‰ *F*_ST_, θπ, and XPEHH.
**Table S13:** Putative selected regions in Asian mouflon‐to‐Chinese native breeds comparison based on 5‰ *F*_ST_, θπ, and XPEHH.
**Table S14:** The overlapping regions in Asian mouflon‐to‐Chinese native breeds based on 5‰ *F*_ST_, θπ, and XPEHH.
**Table S15:** Putative selected regions in Asian mouflon‐to‐improved breeds comparison based on 5‰ *F*_ST_, θπ, and XPEHH.
**Table S16:** The overlapping regions in Asian mouflon‐to‐improved breeds based on 5‰ *F*_ST_, θπ, and XPEHH.
**Table S17:** Putative selected regions in Chinese native breeds‐to‐improved breeds comparison based on 5‰ *F*_ST_, θπ and XPEHH.
**Table S18:** The overlapping regions in Chinese native breeds‐to‐improved breeds based on 5‰ *F*_ST_, θπ, and XPEHH.
**Table S19:** Putative selected regions in fat‐tailed and thin‐tailed sheep population.
**Table S20:** Putative genomic regions under selection for pairwise comparison of five different tail configurations.
**Table S21:** Genome‐wide association study analysis for the tail fat weight trait.
**Table S22:** Genome‐wide association study analysis for the relative weight of tail fat (tail fat weight/carcass weight) trait.
**Table S23:** Overlapping SNPs for the tail fat weight and relative weight of tail fat (tail fat weight/carcass weight) trait.
**Table S24:** Overview of whole genome sequencing data used for selection signature analyses.
**Table S25:** Details of primer sequences used for qRT‐PCR and small interfering RNAs (siRNAs).

## Data Availability

The raw data generated in this study have been deposited in the National Genomics Data Center (https://bigd.big.ac.cn) with the accession codes CRA019589 and CRA019576. The source data and codes used in this study are saved in GitHub (https://github.com/Xiaolong0803/Sheep-epigenome-atlas-and-multi-Omics-analysis). Supplementary materials (figures, tables, scripts, graphical abstract, slides, videos, Chinese translated version, and update materials) may be found in the online DOI or iMeta Science http://www.imeta.science/.
