# Supplementary material for: Comprehensive multi‐tissue epigenome atlas in sheep: A resource for complex traits, domestication, and breeding
Source: Imeta. 2024 Dec 15;3(6):e254. doi: 10.1002/imt2.254 (PMC11683475; doi:10.1002/imt2.254)
Supplement: Supplementary file 1 — Figure S1: Overview of data quality for epigenomic data sets. Figure S2: Principal component analysis (PCA) of different data set types for nine sheep tissues. Figure S3: Characterization of genome‐wide chromatin state across nine tissues. Figure S4: Average gene expression level of different chromatin states across nine tissues. Figure S5: Chromatin states predicated from matched tissue types in sheep, cattle and pigs. Figure S6: Quantitative real‐time reverse transcription PCR (qRT‐PCR) plots in nine tissues of representative tissue‐specific genes across nine tissues. Figure S7: The gene expression, tissue‐specific value (Tau), and GO terms for the three gene groups. Figure S8: Correlation analysis between tissue‐specific gene expression and tissue‐specific enhancers. Figure S9: Tissue‐specific (TS) activate promoters (TssA) and their potential functions across nine tissues. Figure S10: Principal component analysis (PCA) of 364 individuals. Figure S11: Genome‐wide selective signal analysis in different sheep populations. Figure S12: Linkage disequilibrium (LD) analysis of overlapping SNPS for the tail fat weight and relative weight of tail fat (tail fat weight/carcass weight) trait. Figure S13: Functional verification of the BMP2 gene at cell level. [file IMT2-3-e254-s001.docx]

**Supporting information to**

**Comprehensive multi-tissue epigenome atlas in sheep: A resource for complex traits, domestication and breeding**

**Running title:** Construction and application of epigenome atlas in sheep

Deyin Zhang^1#^, Jiangbo Cheng^1#^, Xiaolong Li^1#^, Kai Huang^1^, Lvfeng Yuan^9^, Yuan Zhao^1^, Dan Xu^2^, Yukun Zhang^1^, Liming Zhao^1^, Xiaobin Yang^2^, Zongwu Ma^2^, Quanzhong Xu^1^, Chong Li^2^, Xiaojuan Wang^2^, Chen Zheng^2^, Defu Tang^2^, Fang Nian^8^, Xiangpeng Yue^1^, Wanhong Li^1^, Huibin Tian^1^, Xiuxiu Weng^1^, Peng Hu^7^, Yuanqing Feng^6^, [Peter Kalds](https://pubmed.ncbi.nlm.nih.gov/?term=Kalds+P&cauthor_id=36862946)^5^, Zhihua Jiang^4^, Yunxia Zhao^3^, Xiaoxue Zhang^2^, Fadi Li^1^ and Weimin Wang^1*^

^1^State Key Laboratory of Herbage Improvement and Grassland Agro-ecosystems; Key Laboratory of Grassland Livestock Industry Innovation, Ministry of Agriculture and Rural Afairs; Engineering Research Center of Grassland Industry, Ministry of Education; College of Pastoral Agriculture Science and Technology, Lanzhou University, Lanzhou 730020, China

^2^College of Animal Science and Technology, Gansu Agricultural University, Lanzhou 730070, China

^3^Key Laboratory of Agricultural Animal Genetics, Breeding, and Reproduction of the Ministry of Education, Huazhong Agricultural University, Wuhan 430070, China

^4^Department of Animal Sciences and Center for Reproductive Biology, Washington State University (WSU), Pullman, WA 99164, USA

^5^Yazhouwan National Laboratory, Sanya 572000, China

^6^Department of Genetics, University of Pennsylvania, Philadelphia, PA 19104, USA

^7^Key Laboratory of Exploration and Utilization of Aquatic Genetic Resources, Ministry of Education, Shanghai Ocean University, Shanghai 201306, China

^8^College of Science, Gansu Agricultural University, Lanzhou 730070, China

^9^Lanzhou Veterinary Research Institute, Chinese Academy of Agricultural Sciences (CAAS), Lanzhou 730046, China

^#^These authors contributed equally: Deyin Zhang, Jiangbo Cheng, Xiaolong Li

^*^Correspondence: [wangweimin@lzu.edu.cn](mailto:wangweimin@lzu.edu.cn) (Weimin Wang)

**Supplementary Figures**

**
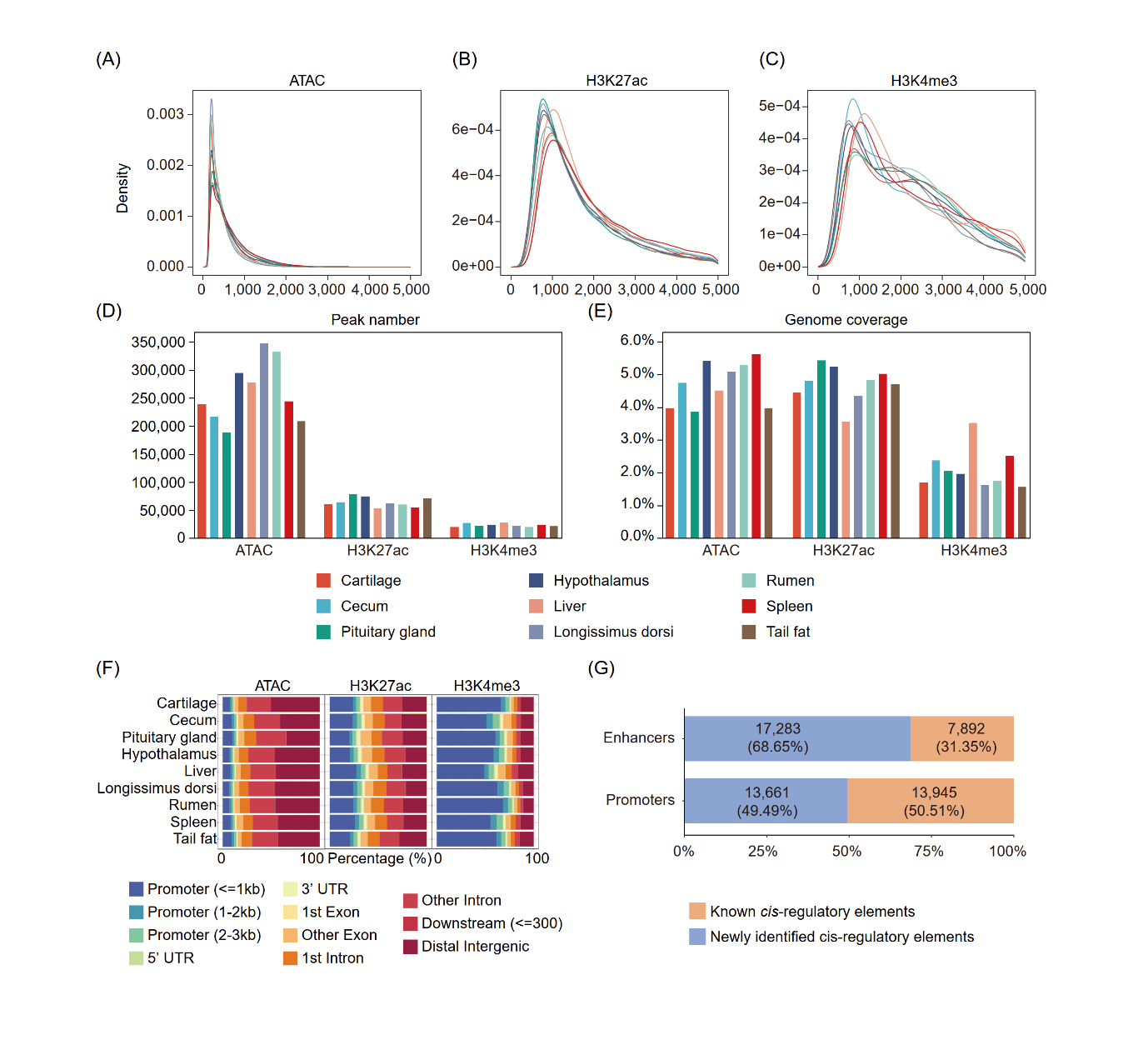
Figure S1 Overview of data quality for epigenomic datasets.** (A-C) Peak length distribution of ATAC-seq, H3K27ac, and H3K4me3 in different tissues. The different color line indicates different tissue. (D-E) The average peak number and genome coverage for epigenetic mark in nine tissues. (F) Distribution and annotation of epigenetic mark peaks across whole genome. (G) Percentages of promoter and enhancer newly detected in this study (blue) and recovered by previously published data from sheep liver tissue (orange). ATAC-seq, Assay for transposase-accessible chromatin using sequencing.


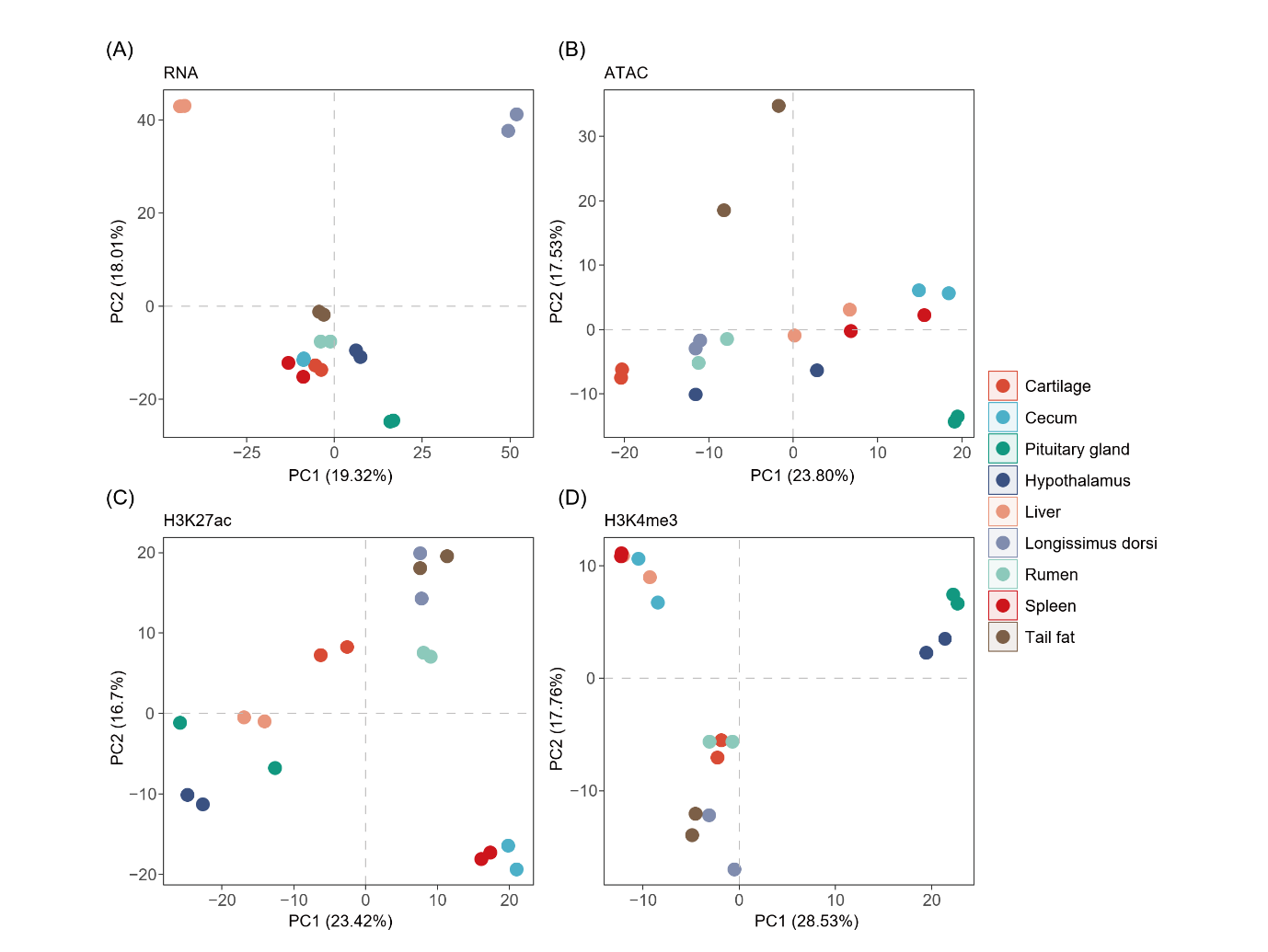


**Figure S2 Principal component analysis (PCA) of different dataset types for nine sheep tissues.** (A) RNA-seq. (B) ATAC-seq. (C) H3K27ac. (D) H3K4me3. Two biological replicates for each tissue. RNA-seq, RNA sequencing.


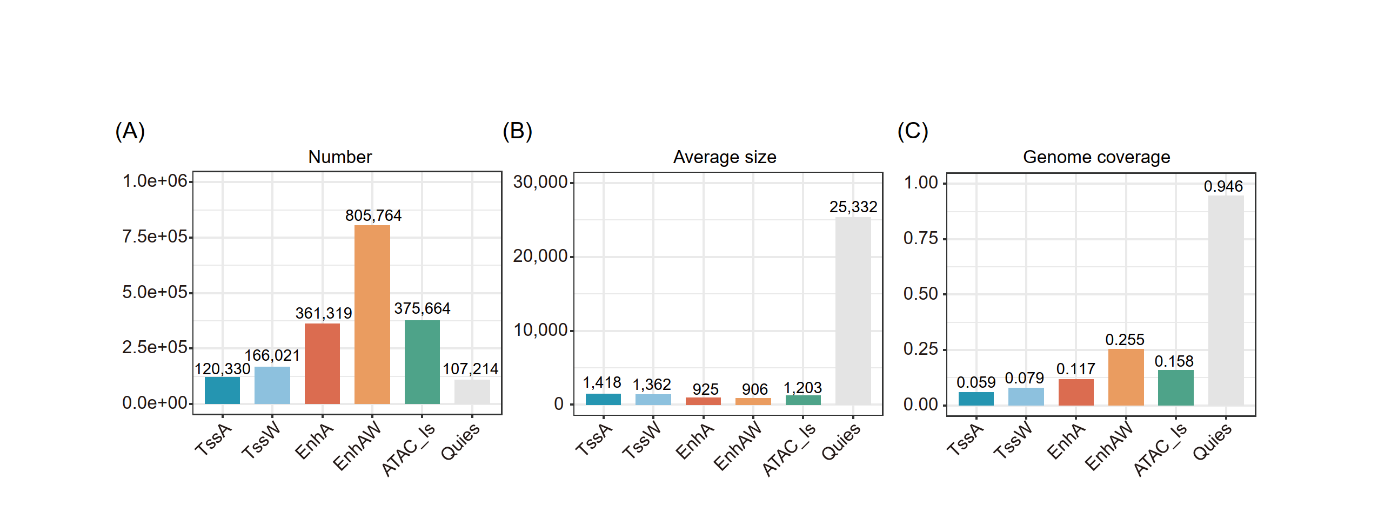
**Figure S3 Characterization of Genome-wide chromatin state across nine tissues.** (A) Number of each chromatin state. (B) Average size of each chromatin state. (C) Genome coverage of each chromatin state. TssA, activate promoters/transcripts, TssW, weak promoters/transcripts, EnhA, strong activate enhancer, EnhAW, weak activate enhancer, ATAC_Is, ATAC island, Quies, quiescent/repression.


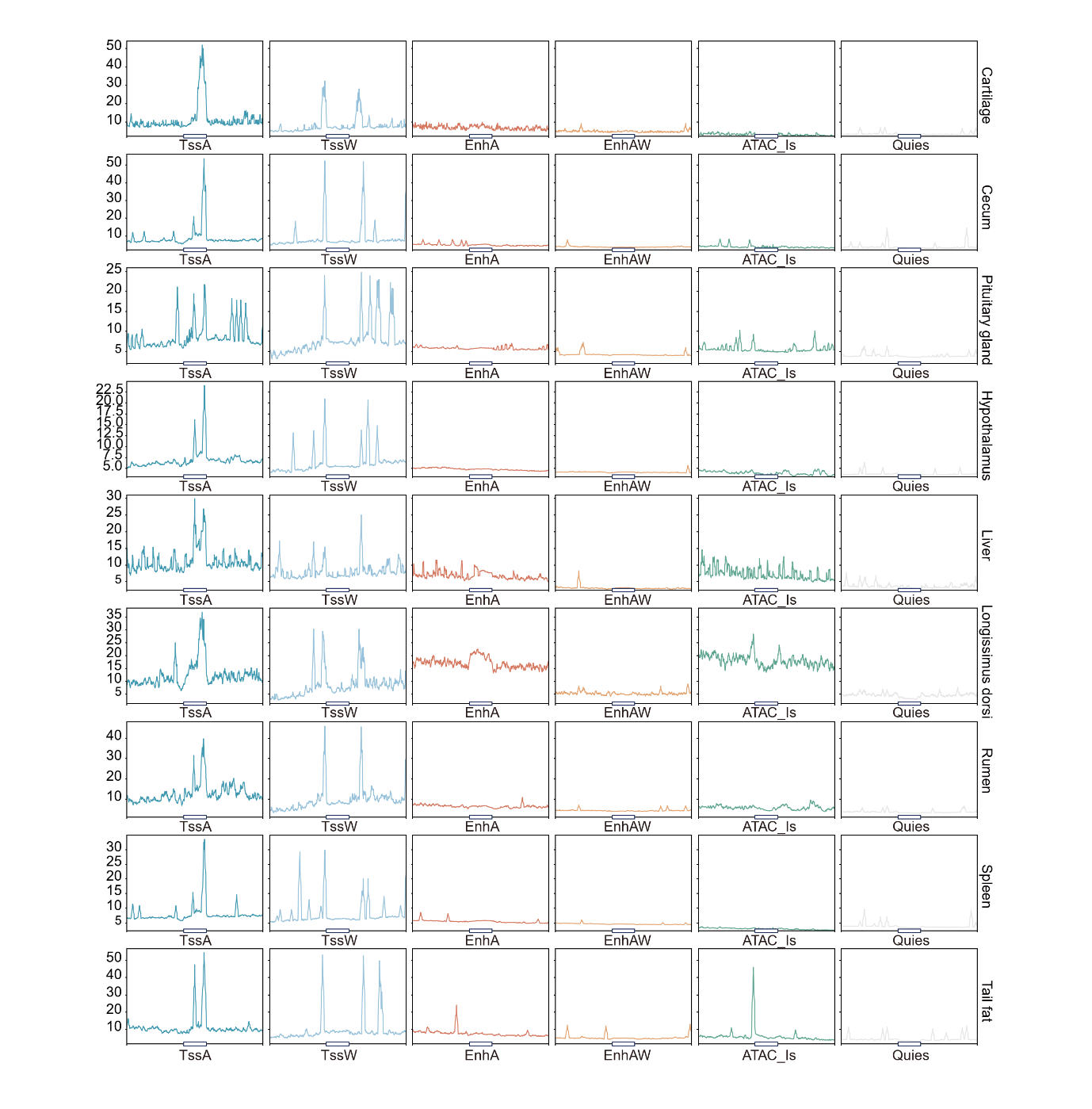
**Figure S4 Average gene expression level of different chromatin states across nine tissues.** TssA, activate promoters/transcripts, TssW, weak promoters/transcripts, EnhA, strong activate enhancer, EnhAW, weak activate enhancer, ATAC_Is, ATAC island, Quies, quiescent/repression.


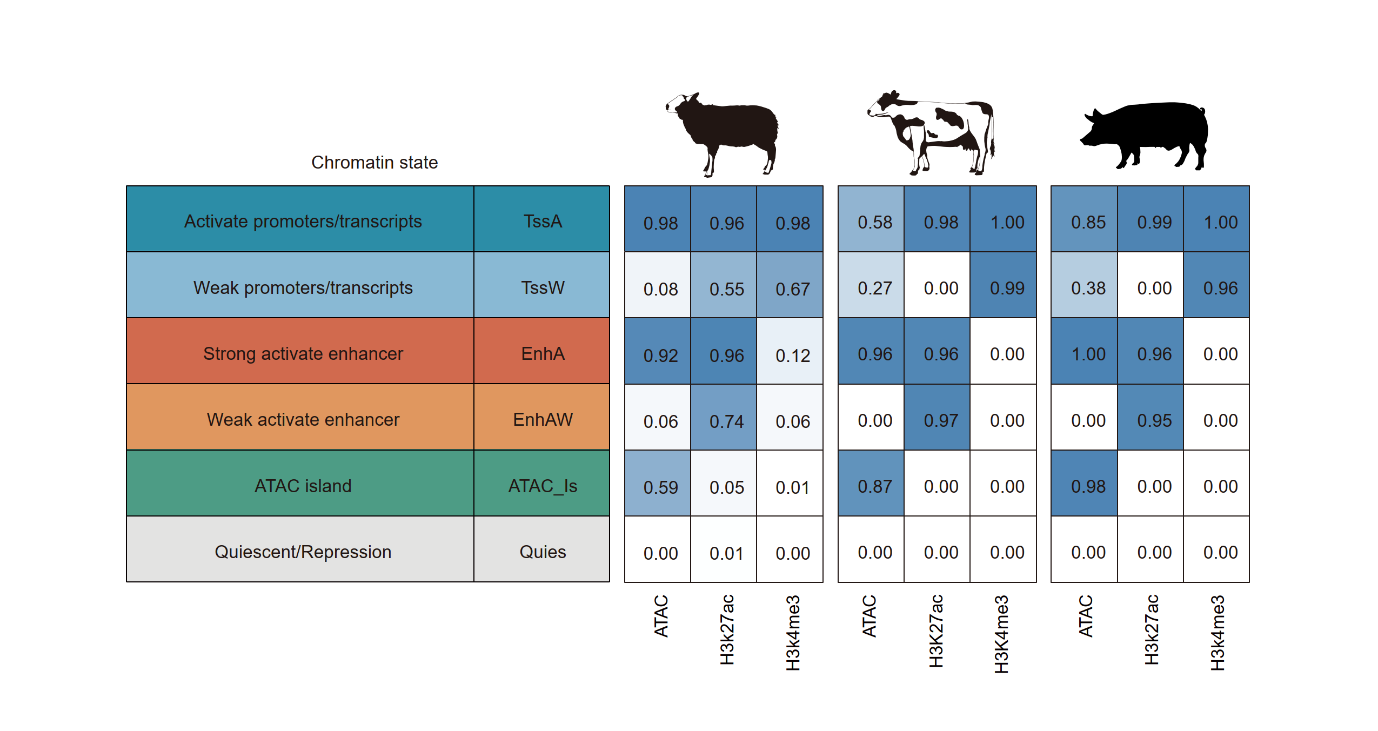
**Figure S5 Chromatin states predicated from matched tissue types in sheep, cattle and pigs.** The figure displays the names, abbreviations, and emission probabilities of the chromatin states in these species. Colors range from white to deep blue, representing emission probabilities from 0 to 1.


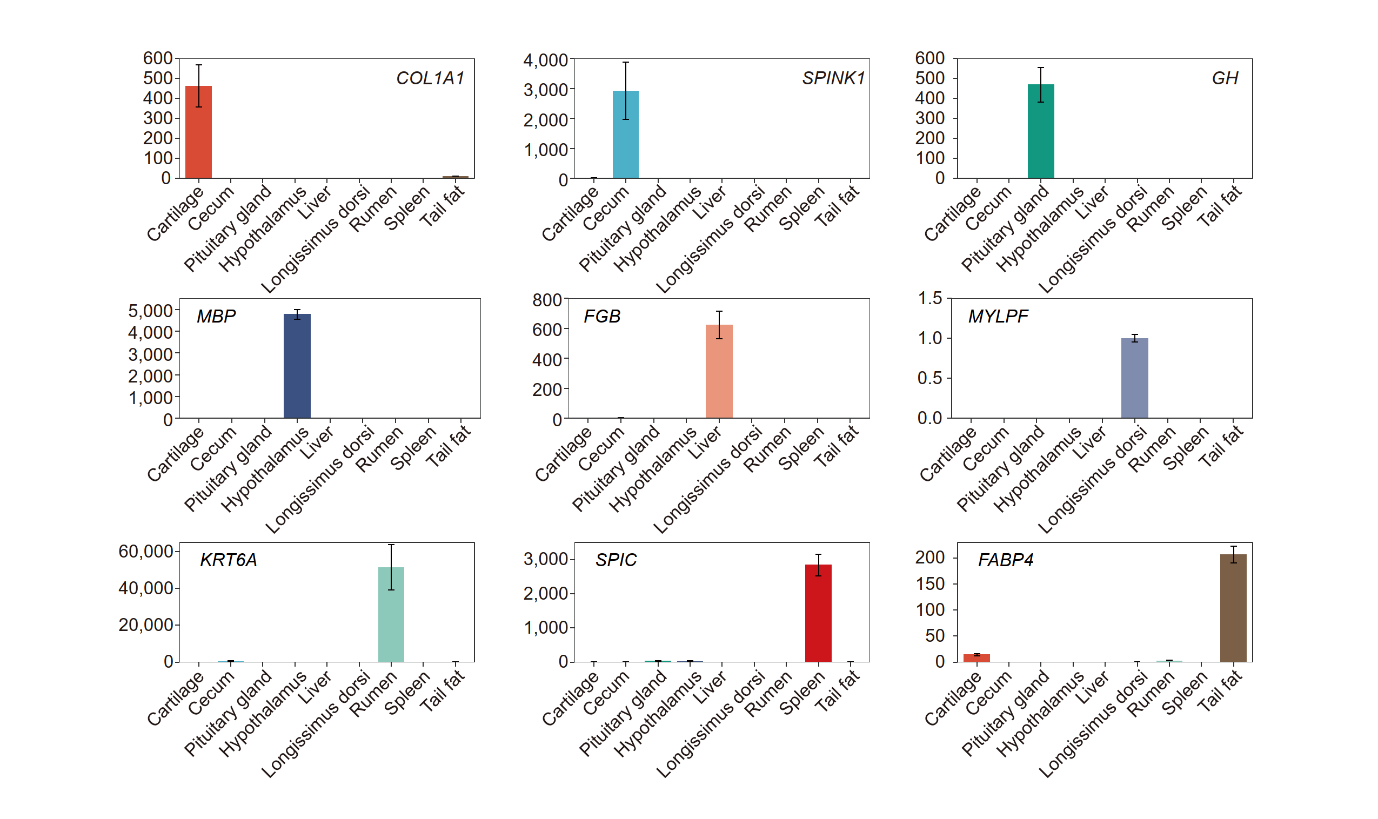
**Figure S6 Quantitative real-time reverse transcription PCR (qRT-PCR) plots in nine tissues of representative tissue-specific genes across nine tissues.** The *UXT* was used as reference gene, data are indicated as means ± standard errors of the means (four technical replicate for each tissue).

**
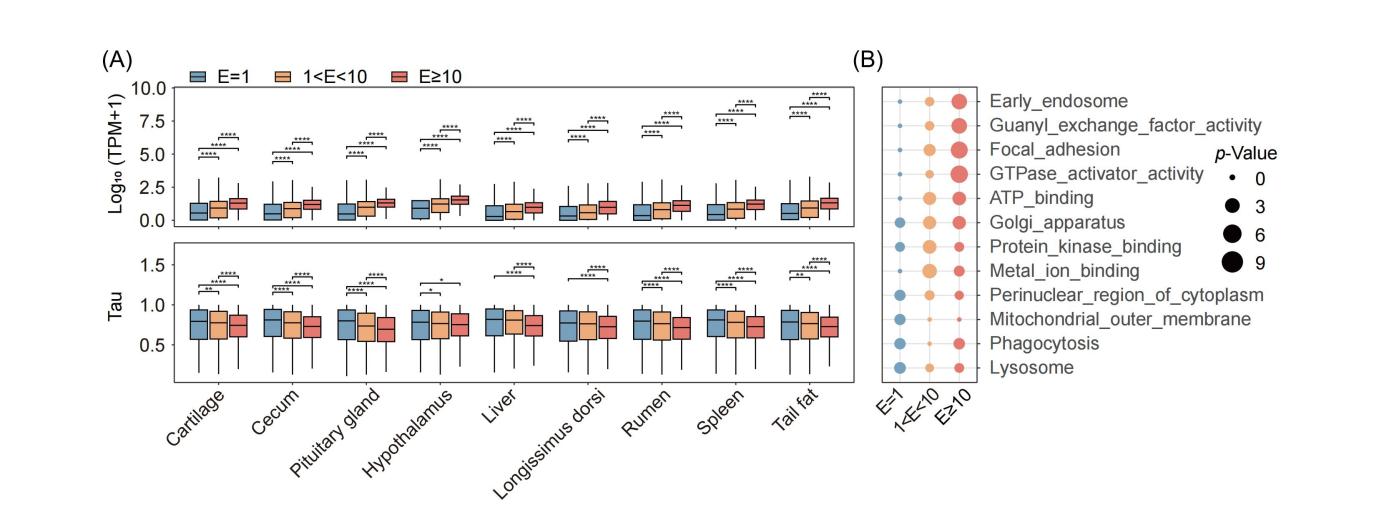
Figure S7 The gene expression, tissue-specific value (Tau) and Gene ontology (GO) terms for the three gene groups.** (A) Expression levels (TPM) and tissue specific values (Tau) of genes overlapping with different numbers of EnhA: E = 1, E = 2–9, and E ≥ 10. Significant differences were identified using the Wilcoxon test. ^*^*p* < 0.05, ^**^*p* < 0.01, ^****^*p* < 0.0001. (B) GO enrichment analysis of genes overlapping with different EnhA numbers in the spleen tissue.


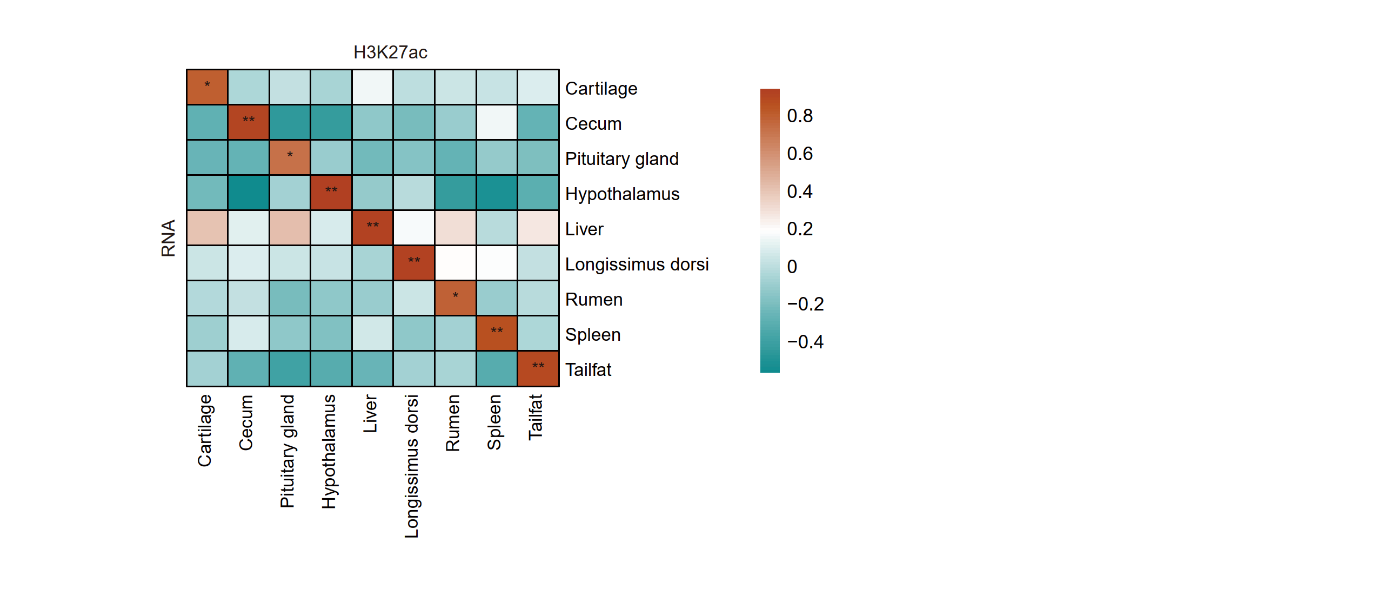
**Figure S8** **Correlation analysis between tissue-specific gene expression and tissue-specific enhancers.** Pearson correlation between tissue-specific gene expression and tissue-specific enhancers, based on transcripts per million (TPM) values for gene expression and reads per kilobase per million mapped reads (RPKM) values for H3K27ac histone marks at enhancer regions. ^*^*p* < 0.05, ^**^*p* < 0.01.

**
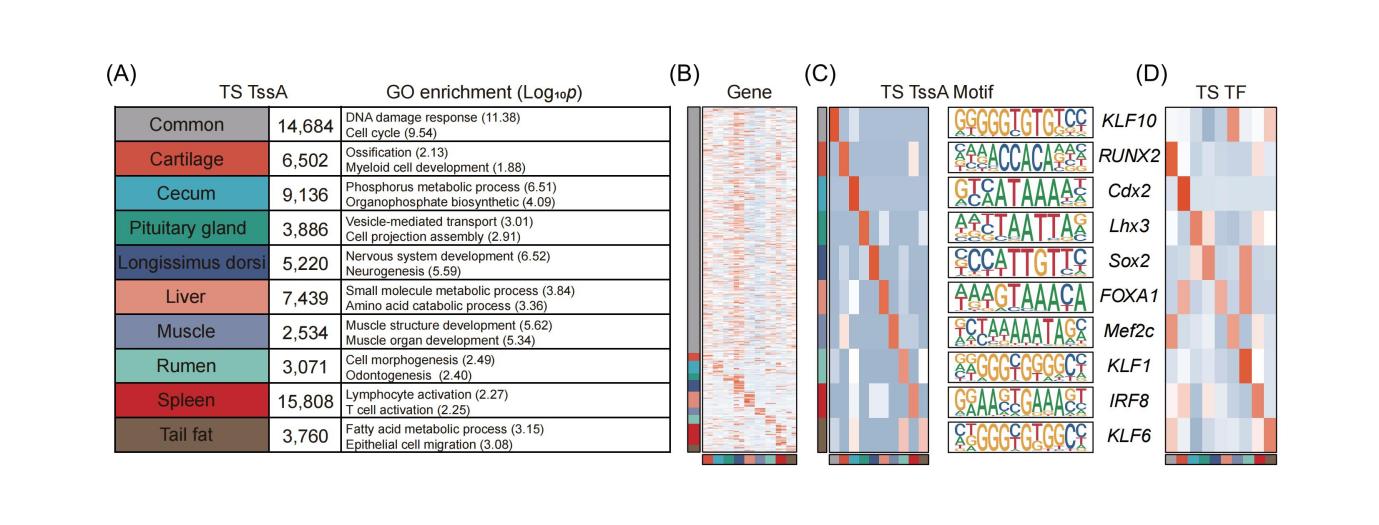
Figure S9 Tissue-specific (TS) activate promoters (TssA) and their potential functions across nine tissues.** (A) Number of tissue-specific TssA, and GO enrichment of genes overlapping tissue-specific TssA. (B) Heatmap of expression patterns of genes overlapping tissue-specific TssA. (C) Motif enrichment in tissue-specific TssA of different tissues, and logos of their sequences. (D) Heatmap of transcription factor (TF) expression patterns corresponding with enriched motifs in different tissue-specific TssA.


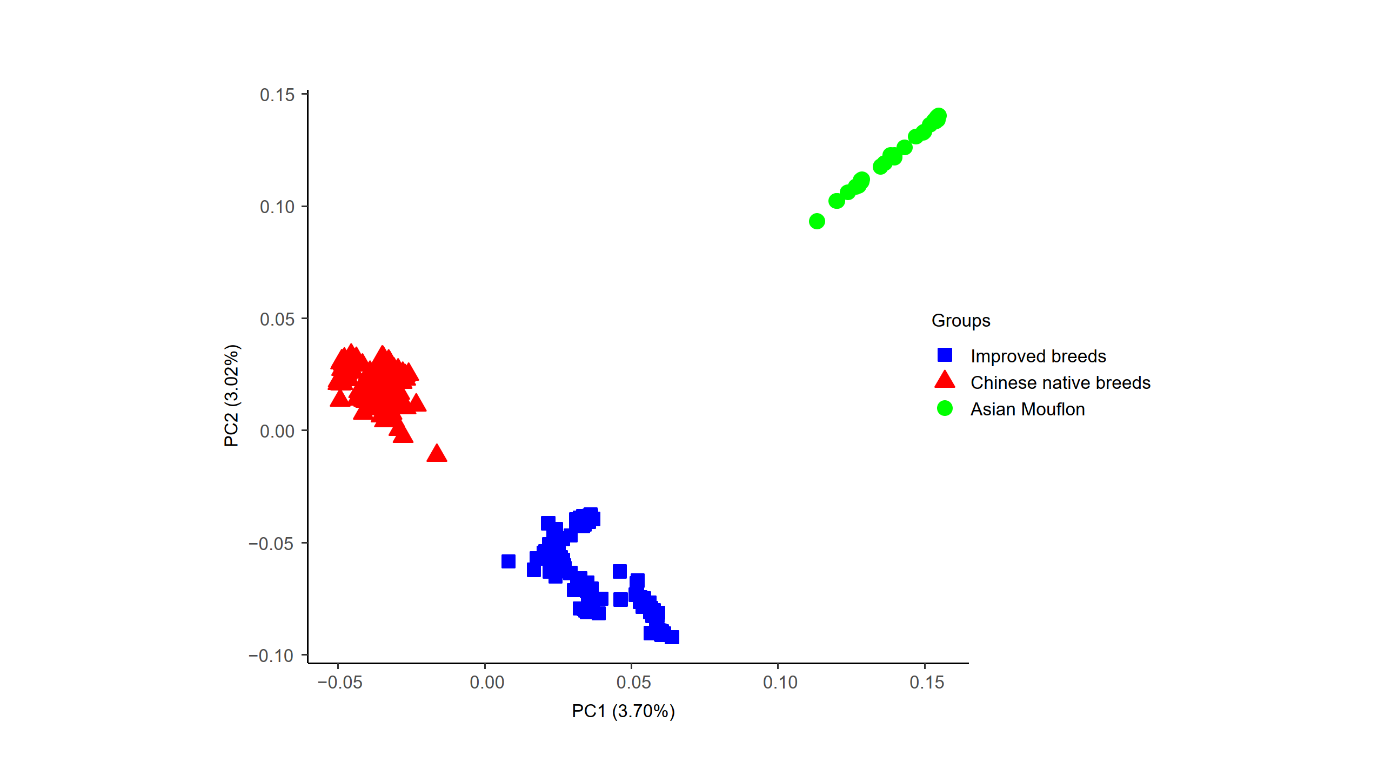
**Figure S10 Principal component analysis (PCA) of 364 individuals.** Plot displayed principal components 1 and 2 (PC1 and PC2) from the PCA of 364 individuals based on the whole-genome sequencing data. The green circles, red triangles and blue squares were indicated Asian mouflon, Chinese native breeds, and improved breeds, respectively.


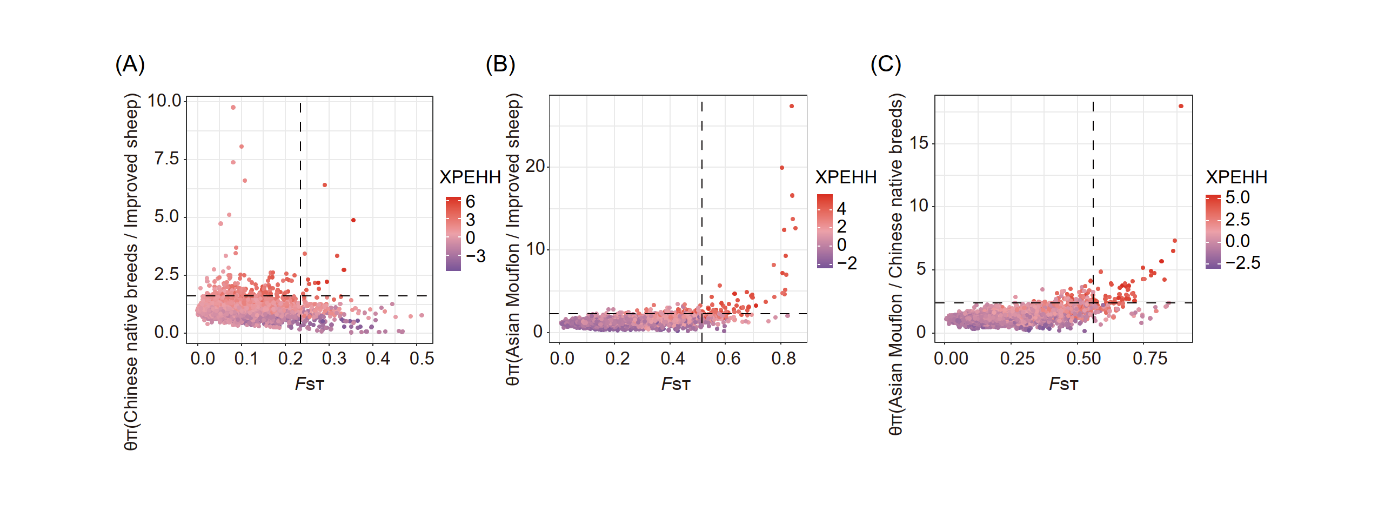
**Figure S11 Genome-wide selective signal analysis in different sheep populations.** (A) Genome-wide selective signals between Chinese native breeds and improved breeds. (B) Genome-wide selective signals between Asian mouflon and improved breeds. (C) Genome-wide selective signals between Asian mouflon and Chinese native breeds. The population genetic differentiation *F_ST_* values are displayed on the x-axis, while the y-axis shows the nucleotide diversity ratio (θπ), with the population extended haplotype homozygosity (XPEHH) values illustrated using a color gradient. The threshold for significant selection signatures was set at the top 5‰ of outliers for each test. The black dashed lines in the upper-right quadrant mark the top 5‰ quantiles of all three statistics.


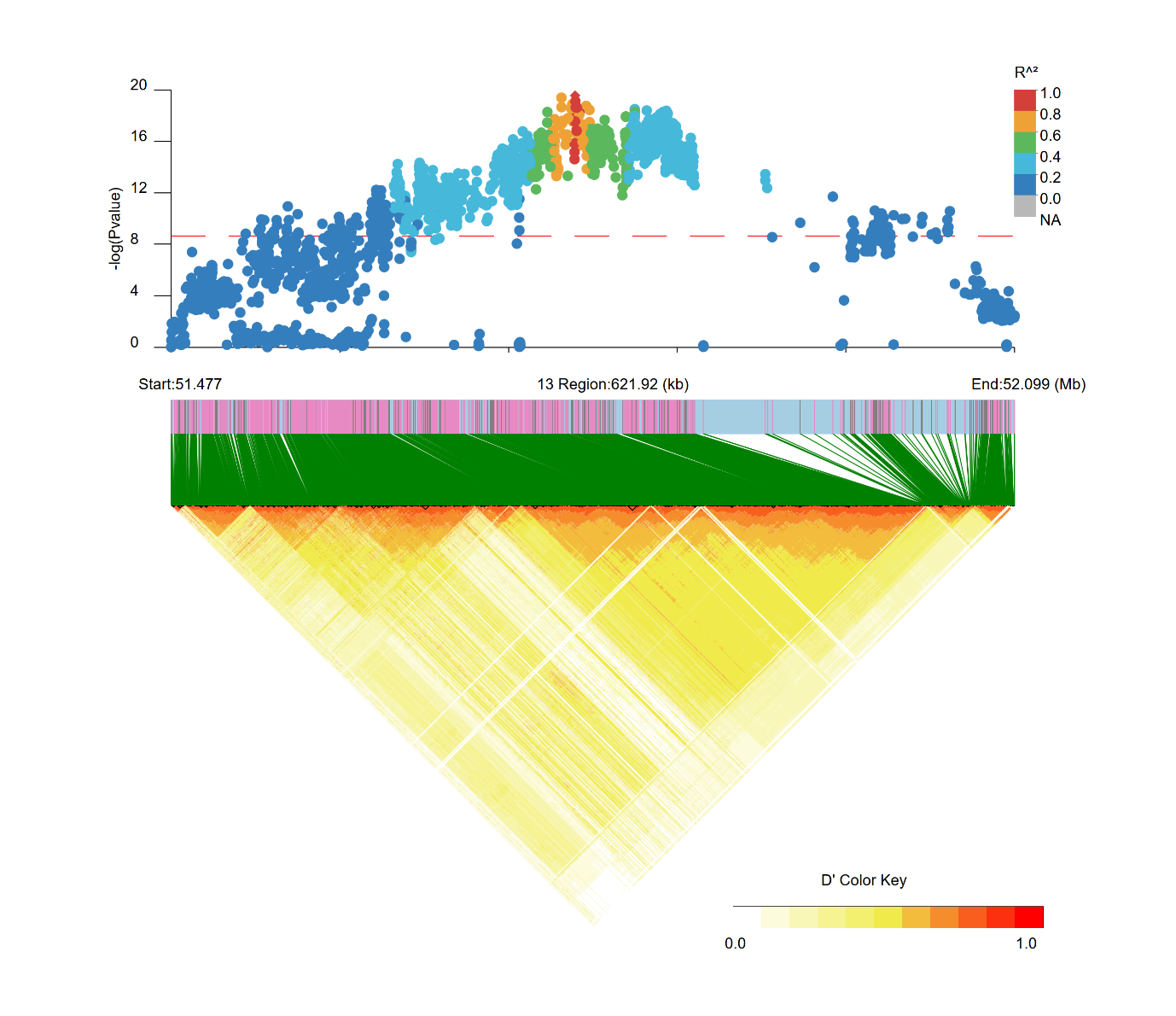
**Figure S12 Linkage disequilibrium (LD) analysis of overlapping SNPS for the tail fat weight and relative weight of tail fat (tail fat weight/carcass weight) trait.** The dashed red line indicates the genome-wide significance threshold [i.e., –log_10_(0.05/total SNPs) = 8.63].


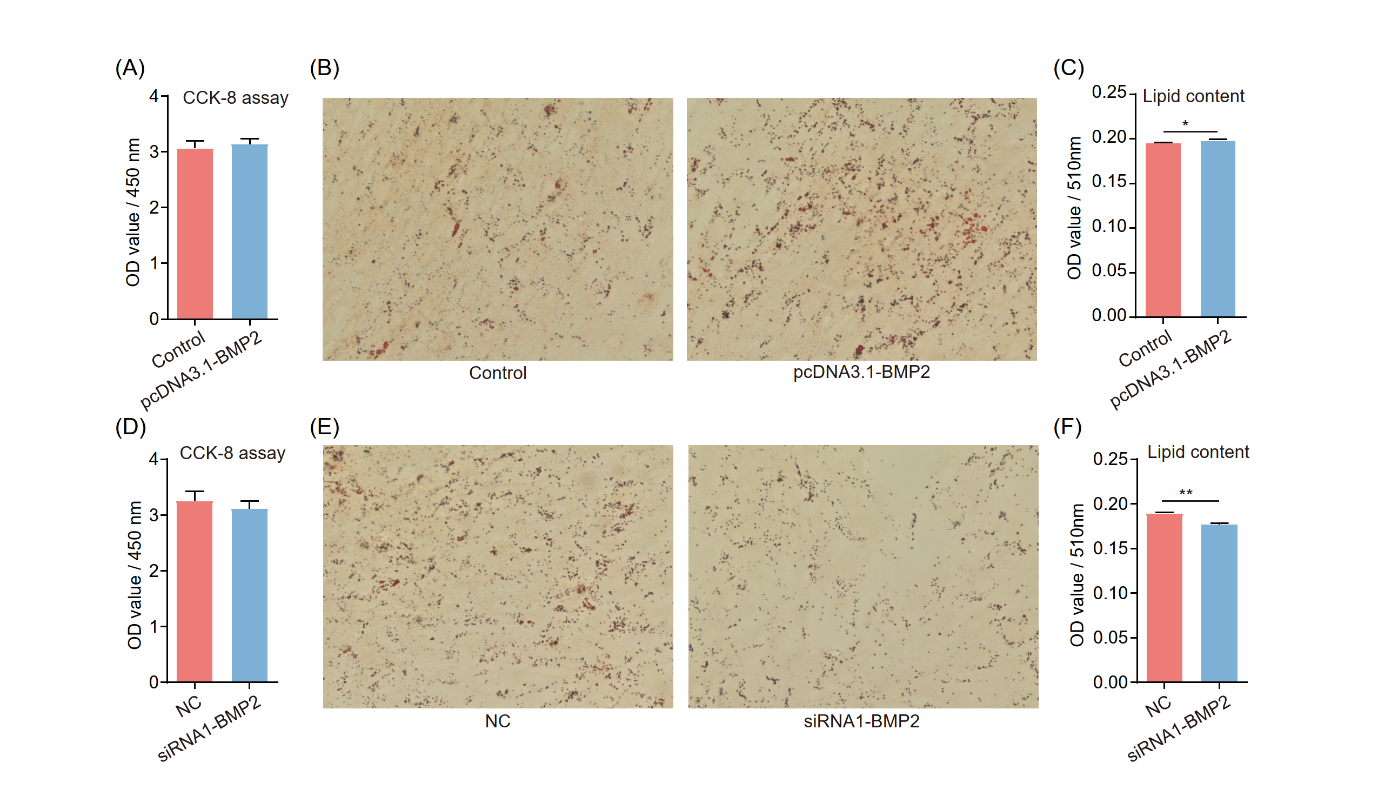
**Figure S13 Functional verification of the *BMP2* gene at cell level.** (A) Cell counting kit-8 (CCK-8) for the proliferation of adipocytes transfected with an empty vector or pcDNA3.1-*BMP2*. (B) Oil red O staining of adipocytes transfected with an empty vector (left) or pcDNA3.1-*BMP2* (right). (C) Lipid content in adipocytes transfected with an empty vector or pcDNA3.1-*BMP2*. (D) CCK8 for the proliferation of adipocytes transfected with a negative control siRNA or siRNA2-*BMP2*. (E) Oil red O staining of adipocytes transfected with negative control siRNA (left) or siRNA2-*BMP2* (right). (F) Lipid content in adipocytes transfected with a negative control siRNA or siRNA2-*BMP2*. Data are indicated as means ± standard errors of the means, differences were analyzed by two-tailed Student’s *t*-test. ^*^*p* < 0.05, ^**^*p* < 0.01.
